# In mice, discrete odors can selectively promote the neurogenesis of sensory neuron subtypes that they stimulate

**DOI:** 10.1101/2024.02.10.579748

**Published:** 2024-02-12

**Authors:** Kawsar Hossain, Madeline Smith, Stephen W. Santoro

**Affiliations:** 1 Department of Pediatrics, Section of Developmental Biology, University of Colorado School of Medicine, Aurora, CO, USA.

## Abstract

In mammals, olfactory sensory neurons (OSNs) are born throughout life, presumably solely to replace neurons lost *via* turnover or injury. This assumption follows from the hypothesis that olfactory neurogenesis is strictly stochastic with respect to neuron subtype, as defined by the single odorant receptor allele that each neural precursor stochastically chooses out of hundreds of possibilities. This hypothesis is challenged by recent findings that the birthrates of a fraction of subtypes are selectively diminished by olfactory deprivation. These findings raise questions about how, and why, olfactory stimuli are required to promote the neurogenesis of some OSN subtypes, including whether the stimuli are generic (e.g., broadly activating odors or mechanical stimuli) or specific (e.g., discrete odorants). Based on RNA-seq and scRNA-seq analyses, we hypothesized that the neurogenic stimuli are specific odorants that selectively activate the same OSN subtypes whose birthrates are accelerated. In support of this, we have found, using subtype-specific OSN birthdating, that exposure to male and musk odors can accelerate the birthrates of responsive OSNs. Collectively, our findings reveal that certain odor experiences can selectively “amplify” specific OSN subtypes, and that persistent OSN neurogenesis may serve, in part, an adaptive function.

## Introduction

Mammalian olfactory epithelia (OE) contain hundreds of distinct olfactory sensory neuron (OSN) subtypes, each of which expresses a single odorant receptor (OR) and thereby detects a distinct set of odorant molecules^1^. The olfactory epithelium is one of a few regions of the mammalian nervous system where neurogenesis occurs throughout life^2–4^. In the hippocampus and olfactory bulb, persistent neurogenesis plays vital roles in learning and memory^5–7^. By contrast, life-long neurogenesis within the mammalian OE is generally assumed to function solely to replace OSNs that are lost due to normal turnover or environmentally induced damage. This assumption follows logically from the prevailing hypothesis that OSN neurogenesis is strictly stochastic with respect to subtype since it is based on the evidently stochastic process of OR choice^8,9^.

Multiple studies have found that the relative quantities of distinct OSN subtypes can be altered by olfactory experience^10–21^. Olfactory deprivation *via* unilateral naris occlusion (UNO), for example, causes changes that include reductions in the representations of a fraction of OSN subtypes^19,21^. Moreover, olfactory enrichment *via* exposure to discrete odors causes changes that include increases in the representations of specific OSN subtypes in mice^15,17^, as well as dramatic increases in sensitivity to specific odors in both rodents and humans^22–27^. Experience-induced changes in the representations of specific OSNs have long been attributed solely to altered OSN lifespan^10,11,16–21,28,29^, in accordance with the hypothesis that OSN neurogenesis is stochastic with respect to subtype, which predicts that the relative birthrates of distinct OSN subtypes should not be affected by olfactory experience. However, a recent study that directly tested this hypothesis by quantifying newborn OSNs of specific subtypes in UNO-treated mice, found that, surprisingly, a fraction of OSN subtypes exhibit selectively reduced birthrates following naris occlusion^30^. Moreover, the subtypes whose birthrates are diminished by naris occlusion do not show unusual levels of baseline activity compared to subtypes whose birthrates are unaffected, demonstrating that reduced OSN activity alone is insufficient to diminish the birthrates of specific subtypes^30^. Rather, a fraction of OSN subtypes appear to have a special capacity to undergo changes in birthrates in accordance with the level of olfactory stimulation. Because naris occlusion reduces exposure to potentially thousands of odors, as well as mechanical stimuli, and may cause additional physiological changes^31^, these findings raise questions about the nature of the stimuli that affect neurogenesis. Addressing these questions is crucial for understanding the mechanism and function of stimulation-dependent neurogenesis.

Here we sought to identify the stimuli that are required to promote the neurogenesis of specific OSN subtypes. We envisioned that these could be either non-specific stimuli unrelated to the subtypes whose birthrates are reduced by deprivation (e.g., generic odors, mechanical stimuli, or other physiological effects of UNO) or, alternatively, discrete odorants that selectively stimulate the same subtypes whose birthrates are affected. We envisioned that distinguishing between these possibilities would provide insights into the mechanism and function of this process. If the neurogenic stimuli are non-specific, this would imply a generic mechanism and, perhaps, a homeostatic function. By contrast, if the stimuli are discrete odorants that selectively stimulate the same OSN subtypes whose birthrates are affected, this would imply a highly specific mechanism in which exposure to certain odors can “amplify” OSN subtypes responsive to those odors. The latter outcome would also suggest that OE neurogenesis serves, in part, an adaptive function.

Findings from previous studies led us to favor the hypothesis that the neurogenic stimuli comprise discrete odorants that selectively activate the same OSN subtypes whose birthrates are accelerated. One prediction of this hypothesis is that the extent to which naris occlusion reduces the birthrates of specific OSN subtypes should vary depending on the odor environment to which animals are exposed. In support of this, open-side biases in the birthrates of specific OSN subtypes were found to vary depending on whether a mouse was in the nursing (P14) or post-weaning stage (P28) at the time of birthrate assessment^30^. A second prediction is that exposure of non-occluded mice to specific odors should selectively increase the representation of subtypes responsive to those odors within the OE. Consistent with this, a previous study identified several OSN subtypes that exhibit greater representation in mice housed in the presence of sex-specific odors, compared to the absence, until six months of age^17^. These findings were corroborated by a complementary study from a different group^32^. Interestingly, several of the affected OSN subtypes were also found to be selectively responsive to male or female odors^17^, suggesting that exposure to components of sex-specific odors results in greater representations of these OSNs. These changes had been hypothesized to be caused by subtype-selective lengthening of the lifespans of OSNs of specific subtypes in the presence of sex-specific odors^17^. However, in light of recent findings that naris occlusion selectively reduces the birthrates of specific OSN subtypes^30^, we considered the alternative explanation that sex-specific odor-dependent increases in the representations of specific OSN subtypes are caused by altered rates of neurogenesis. Here we present evidence that discrete odors can selectively accelerate the birthrates of OSN subtypes that they stimulate. These findings support the hypothesis that the function of lifelong OSN neurogenesis is not strictly homeostatic, but also enables adaptive changes to the composition of the OSN population. These findings may also have mechanistic relevance to intriguing and unexplained observations in both rodents and humans that exposure to specific odors can dramatically increase sensitivity to them^22–27^.

## Results

### Bulk and single-cell RNA-seq analyses reveal that exposure to male odors is associated with increased quantities of newborn OSNs of musk-responsive subtypes.

To investigate whether discrete odorants can selectively accelerate the birthrates of OSN subtypes that they stimulate, we sought to identify subtypes that show evidence of a capacity for stimulation-dependent neurogenesis and for which stimulating odors have been identified. A challenge of this approach is that odorant ligands remain unidentified for most OSN subtypes^33^, including all subtypes previously found to undergo stimulation-dependent neurogenesis^30^. To overcome this, we wondered whether candidate subtypes might be identified among those previously found to be more highly represented in mice exposed to odors emitted by a particular sex^17^. Of these subtypes, Olfr235 *(Or5an11)* appeared especially intriguing, as it was observed to be more highly represented in mice exposed to male conspecifics (sex-separated males; sex-combined males and females) compared to mice isolated from them (sex-separated females)^30,32^ (Supplementary Fig. 1). Moreover, Olfr235 OSNs showed selective responsivity to male-specific odors^17^ (Supplementary Fig. 1). Taken together, these findings suggested that exposure of mice to male odors leads to an increase in the representation of subtype Olfr235 within the OE. Intriguingly, Olfr235 belongs to a group of related ORs that include Olfr1440 *(Or5an6)*, Olfr1437 *(Or5an1b)*, Olfr1431 *(Or5an9)*, and Olfr1434 *(Or5an1)*, members of which detect musk-like odors with varying levels of sensitivity^34,35^. Notably, like Olfr235, all other musk responsive ORs were found to exhibit higher transcript levels in the OEs of mice exposed to males compared to mice isolated from males (except Olfr1434, whose transcript levels were too low to be accurately assessed)^17^ (Supplementary Fig. 1). Accordingly, fluorescent *in situ* hybridization (FISH) analyses of a subset of these ORs, Olfr235 and Olfr1437, confirmed that the elevated transcript levels observed in mice exposed to male conspecifics reflected greater OSN quantities^17^. By contrast, Olfr912 *(Or8b48)* and Olfr1295 (*Or4k45)*, which detect the male-specific non-musk odors 2-sec-butyl-4,5-dihydrothiazole (SBT) and (methylthio)methanethiol (MTMT), respectively^32^, exhibited lower representation and/or transcript levels in mice exposed to male odors^17,32^ (Supplementary Fig. 1), possibly reflecting reduced survival due to overstimulation. Taken together, these findings indicate that OSN subtypes responsive to musk odors are selectively increased in their representation upon exposure to male mice, conceivably due to accelerated neurogenesis in mice exposed to male odors.

**Figure 1.**
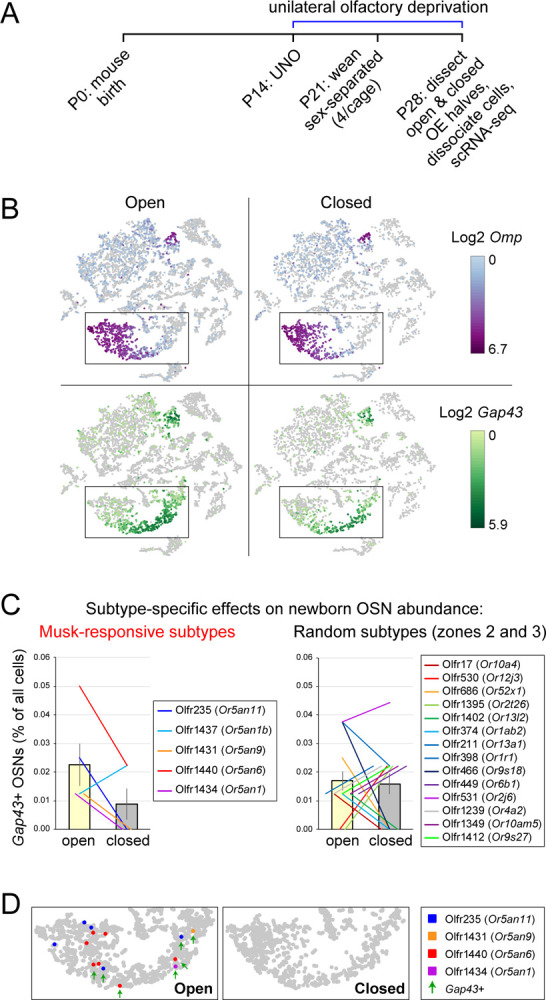
scRNA-seq analysis of OEs from UNO-treated male mice shows reduced quantities of newborn OSNs of musk-responsive subtypes on the closed side of the OE relative to the open. A. scRNA-seq datasets that were used to quantify newborn OSNs of musk-responsive subtypes were generated from the open and closed side of the OE of a mouse that was UNO-treated at P14 and sacrificed at P28^30^. B. *t*-SNE plot representation of the scRNA-seq datasets corresponding to the open (*left*) and closed (*right*) sides of the OE, showing *Omp* (mature OSNs, *top*) and *Gap43* (immature OSNs, *bottom*) expression. C. Quantification of individual (lines) and average (bars) percentages of the OE cell population represented by immature (*Gap43*+) OSNs of musk-responsive subtypes (*left*) or randomly chosen zone 2/3 subtypes (*right*) within the open and closed datasets. D. Identification of OSNs of the 4 musk-responsive subtypes that are more highly represented within the open dataset compared to the closed. *Green arrows*: *Gap43+* OSNs. See also Supplementary Fig. 2.

To begin to investigate whether musk-responsive OSN subtypes undergo accelerated neurogenesis in the presence of male odors, we used an scRNA-seq dataset comprising the transcriptomes of single cells dissociated from the open and closed sides of an olfactory epithelium (OE) of a male mouse that had been UNO-treated at P14 and dissected at P28 (Fig. 1A)^30^. Within this dataset, newborn (*Gap43*+) OSNs of subtypes that undergo stimulation-dependent neurogenesis were previously found to be more highly represented on the open side of the OE compared to the closed side^30^. Interestingly, our identification of newborn OSNs of five known musk-responsive subtypes within this dataset revealed a 2.5-fold greater representation in the open-side scRNA-seq library compared to the closed (Fig. 1B, C-*left*; Supplementary Fig. 2A). We observed a similar open-side bias for newborn Olfr1419 (*Or10q3*) OSNs (Supplementary Fig. 2B-*left*), a subtype with unknown odor responsivity that was also previously found to have a higher overall representation in mice exposed to male odors (Supplementary Fig. 1B) and a reduced representation following olfactory deprivation^17^. By contrast, we observed that newborn OSNs of 14 randomly chosen subtypes located in the same region of the OE where musk-responsive subtypes reside (canonical zones 2 and 3)^36^ comprise a nearly equal proportion of cells on the open side relative to the closed (1.07-fold difference) (Fig. 1C-*right*), as do newborn OSNs of 7 known stimulation-independent subtypes (0.73-fold difference) (Supplementary Fig. 2B-*right*). Differences in the representation of newborn and mature OSNs of subtypes Olfr235, Olfr1440, Olfr1431, and Olfr1434 on the open versus the closed sides are observable in the t-distributed Stochastic Neighbor Embedding (t-SNE) plots of the corresponding cell populations (Fig. 1D). Taken together, these data are consistent with the hypothesis that subtypes of OSNs responsive to musk odors undergo stimulation-dependent neurogenesis.

### Olfactory deprivation reduces quantities of newborn OSNs of musk-responsive subtypes in male mice.

**Figure 2.**
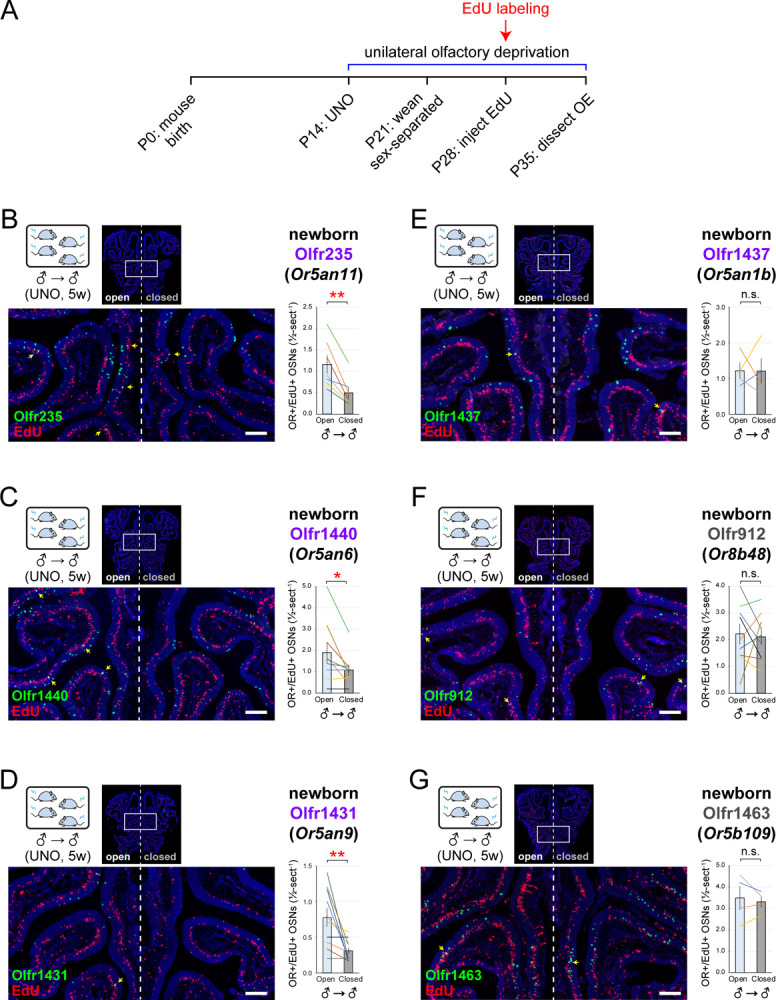
Histological analyses confirm that olfactory deprivation reduces quantities of newborn OSNs of musk-responsive subtypes in male mice. A. Experimental timeline for the analysis of open-side biases in quantities of newborn OSNs of musk-responsive and control subtypes in male mice that were UNO treated at P14, weaned sex-separated at P21, EdU-labeled at P28, sacrificed at P35, and analyzed *via* OR-specific RNA-FISH and EdU staining. B-G. Representative images (*left*) and quantification (*right*) of newborn OSNs (OR+/EdU+) of subtypes Olfr235 (B), Olfr1440 (C), Olfr1431 (D), Olfr1437 (E), Olfr912 (F), or Olfr1463 (G) within sections of OEs from UNO-treated and EdU-labeled male mice that were exposed to themselves at the time of EdU labeling (♂ → ♂). OR+/EdU+ cells (*yellow arrows*) are newborn OSNs of the indicated subtypes. Scale bars: 150 μm. Musk-responsive and control subtypes are labeled in purple and gray type, respectively. Each line represents a distinct mouse (*n* = 4–10 mice/OSN subtype).

To confirm that musk-responsive subtypes undergo stimulation-dependent changes in newborn OSN quantities, we employed an established histological assay in which EdU-birthdated OSNs of specific subtypes are quantified in UNO-treated mice *via* EdU staining and OR-specific FISH^30,37^. Using this approach, we quantified newborn OSNs of musk-responsive subtypes on the open and closed sides of the OEs of male mice that had been UNO-treated at P14, EdU-injected at P28, and dissected at P35 (Fig. 2A). Consistent with our findings *via* scRNA-seq, histological analyses revealed that newborn OSNs of subtypes Olfr235, Olfr1440, and Olfr1431 are 2.3-fold (*p* = 0.003), 1.8-fold (*p* = 0.03), and 2.5-fold (*p* = 0.009) more abundant on the open side of the OE compared to the closed in juvenile male mice (Fig. 2B–D). Moreover, all three subtypes exhibited open-side biases in total OSN quantities (*p* < 0.003) (Supplementary Fig. 3A–C, *left*). Also consistent with the scRNA-seq data, subtype Olfr1437 exhibited no significant open-side biases in either newborn (1.01-fold; *p* = 0.99) (Fig. 2E) or total OSN quantities (1.2-fold; *p* = 0.09) (not shown). Likewise, two non-musk-responsive control subtypes, Olfr912 and Olfr1463 (*Or5b109*), showed no significant open-side biases in newborn OSN quantities (1.05-fold for both; *p* > 0.6) (Fig. 2F, G). However, consistent with previous findings^17^, subtype Olfr912 exhibited a 1.5-fold higher abundance of total OSNs on the closed side (*p* = 0.0002) (Supplementary Fig. 3D, *left*), likely reflecting a lengthening of OSN lifespan for this subtype due to protection from overstimulation following olfactory deprivation^30,32^. By contrast, total OSNs of subtype Olfr1463 exhibited no significant bias (*p* = 0.99) (Supplementary Fig. 3E, *left*). Taken together, these findings further support the hypothesis that a subset of musk responsive OSN subtypes undergo stimulation-dependent neurogenesis.

### Olfactory deprivation-induced reductions in quantities of newborn Olfr235 OSNs depend on exposure to male odors.

**Figure 3.**
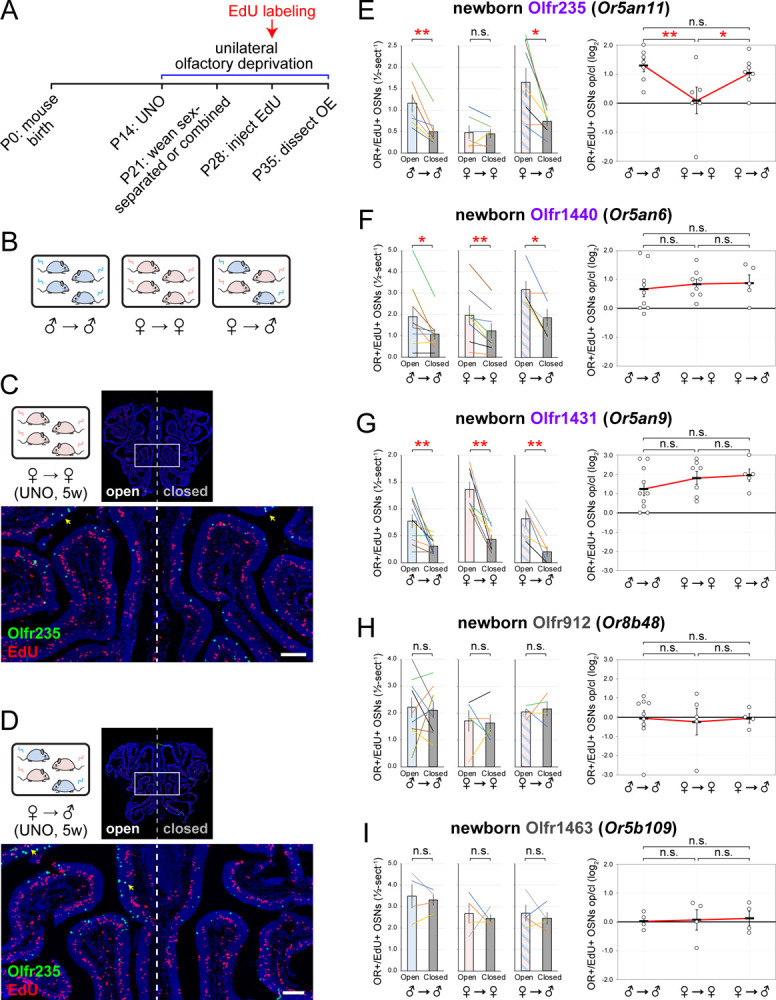
Olfactory deprivation-induced reductions in quantities of newborn Olfr235 OSNs depend on exposure to male odors. A, B. Experimental timeline and schematic for the analysis of open-side biases in quantities of newborn OSNs of musk-responsive and control subtypes in male mice that were UNO treated at P14, weaned sex-separated or sex-combined at P21, EdU-labeled at P28, sacrificed at P35, and analyzed *via* OR-specific FISH and EdU staining. C, D. Representative images of OE sections stained for EdU and Olfr235 from UNO-treated female mice exposed to themselves (♀ → ♀) (C) or to male mice (♀ → ♂) and (D) at the time of EdU-labeling. OR+/EdU+ cells (*yellow arrows*) are newborn Olfr235 OSNs. Scale bars: 150 μm. E-I. Quantification (*left*) and UNO effect sizes (*right*) of newborn OSNs (OR+/EdU+) of subtypes Olfr235 (E), Olfr1440 (F), Olfr1431 (G), Olfr912 (H), and Olfr1463 (I) within OEs of UNO-treated male mice exposed to themselves (♂ → ♂), female mice exposed to themselves (♀ → ♀), or female mice exposed to male mice (♀ → ♂) at the time of EdU labeling. Musk-responsive and control subtypes are labeled in purple and gray type, respectively. Each line or circle represents a distinct mouse (*n* = 4–10 mice per OSN subtype and condition). See also Supplementary Fig. 3.

Having determined that subtypes Olfr235, Olfr1440, and Olfr1431 exhibit open-side biases in the quantities of newborn OSNs in UNO-treated male mice, we next investigated whether these biases require exposure to odors emitted specifically by male mice. If so, we predicted that open-side biases would be attenuated in mice isolated from male odors. To test this, we quantified newborn OSNs of the three subtypes in UNO-treated *female* mice that were separated from their male littermates at weaning (P21) and thus exposed only to female littermates at the time of EdU treatment (P28) (Fig. 3A, B). Strikingly, unlike their male counterparts, sex-separated female mice exhibited no significant bias in newborn Olfr235 OSN quantities (1.1-fold; *p* = 0.8) (Fig. 3C, E-*left*), a result also observed for total OSN quantities of this subtype (1.2-fold; *p* = 0.1) (Supplementary Fig. 3A-*left*). To compare the effects of UNO between experimental groups, we defined a UNO effect size as the log_2_ (open/closed) ratio for the quantity of OSNs of a specific subtype and category (newborn or total). Using this definition, we found that the average UNO effect size for quantities of newborn Olfr235 OSNs was 14-fold larger (*p* = 0.014) in sex-separated males compared to females (Fig. 3E-*right*), and 2.6-fold larger for total Olfr235 OSNs (*p* = 0.04; Supplementary Fig. 3A-*right*). These data indicate that open-side biases in newborn Olfr235 OSN quantities either require exposure to a male-emitted odor or are intrinsic to males. If the observed biases are male-odor driven, we would expect female mice housed with males to exhibit open-side biases similar in magnitude to those observed in males. To test this, we quantified newborn and total Olfr235 OSNs in juvenile females housed with their male littermates at weaning (Fig. 3A, B). Strikingly, females co-housed with males exhibited a 2.2-fold (*p* = 0.015) greater quantity of newborn Olfr235 OSNs (Fig. 3E-*left*) and a UNO effect size 11-fold greater (*p* < 0.037) than that observed for sex-separated females but not significantly different than that observed for sex-separated males (0.8-fold difference; *p* = 0.21) (Fig. 3E-*right*). Analogous open-side biases and differences in UNO effect sizes were observed for total Olfr235 OSN quantities (Supplementary Fig. 3A). These results indicate that stimulation-dependent changes in newborn Olfr235 OSN quantities require exposure to male-emitted odors.

In contrast to subtype Olfr235, subtypes Olfr1440 and Olfr1431 exhibited similar levels of open-side bias in newborn OSN quantities between experimental groups. For subtype Olfr1440, open-side biases in newborn OSN quantities were 1.8-fold (p = 0.03) in sex-separated males, 1.6-fold (p = 0.003) in sex-separated females, and 1.7-fold (p = 0.03) in sex-combined-females (Fig. 3F-left). For subtype Olfr1431, open-side biases in newborn OSN quantities were 2.5-fold (*p* = 0.009), 3.2-fold (*p* = 0.002), and 3.8-fold (*p* = 0.009), respectively (Fig. 3G-*left*). Accordingly, the UNO effect sizes for both Olfr1440 and Olfr1431 subtypes were not significantly different between the three experimental groups for newborn (Fig. 3F, G-*right*) or total OSNs (Supplementary Fig. 3B, C-*right*) (*p* > 0.3). As expected, two negative control subtypes, Olfr912 and Olfr1463, exhibited no significant open-side biases in newborn OSN quantities in any experimental group (*p* > 0.64) (Fig. 3H, I), while quantities of total Olfr912 OSNs exhibited closed-side biases under all conditions (Supplementary Fig. 3D).

Taken together, these findings indicate that open-side biases in quantities of newborn Olfr235 OSNs require exposure to an odor that is emitted specifically by male mice, at least at the juvenile stage. By contrast, open-side biases in quantities of newborn Olfr1440 and Olfr1431 OSNs may be driven by an odor either emitted by both male and female mice at this stage, or by another environmental source.

### Exposure to adult mice intensifies deprivation-induced reductions in quantities of newborn Olfr1431 OSNs.

**Figure 4.**
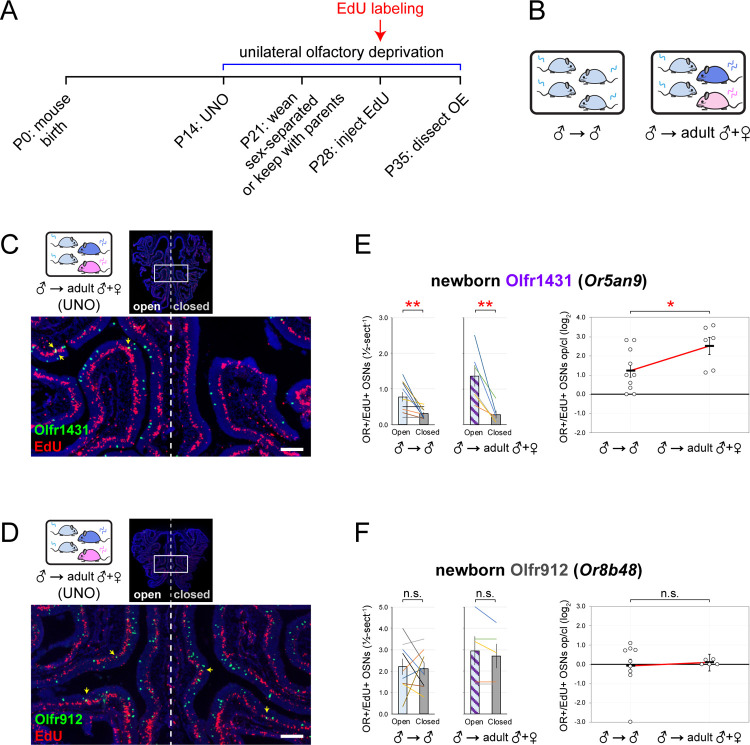
UNO-induced changes in newborn Olfr1431 OSN quantities are intensified by exposure adult mice. A, B. Experimental timeline and schematic for the analysis of open-side biases in quantities of newborn OSNs of specific subtypes in male mice that were UNO treated at P14, weaned sex-separated (♂ → ♂) or kept with parents (♂ → adult ♀ + ♂) at P21, EdU-labeled at P28, sacrificed at P35, and analyzed *via* OR-specific FISH and EdU staining. C, D. Representative images of OE sections stained for EdU and Olfr1431 (C) or Olfr912 (D) from UNO-treated male mice exposed to their parents (♂ → adult ♀ + ♂) at the time of EdU-labeling. OR+/EdU+ cells (yellow arrows) are newborn OSNs of the indicated subtypes. Scale bars: 150 μm. E, F. Quantification (left) and UNO effect sizes (right) of newborn OSNs (OR+/EdU+) of subtypes Olfr1431 (E) or Olfr912 (F) within OEs of UNO-treated male mice exposed to themselves (♂ → ♂) or their parents (♂ → adult ♀ + ♂) at the time of EdU labeling. Musk-responsive and control subtypes are labeled in purple and gray type, respectively. Each line or circle represents a distinct mouse (*n* = 5–10 mice per OSN subtype and condition). See also Supplementary Fig. 4.

Observations that exposure to male odors is required for stimulation-dependent changes in the quantities of newborn Olfr235 but not Olfr1440 or Olfr1431 OSNs were intriguing considering that ORs of all three subtypes show male-biased expression in mice housed sex-separated until 6 months of age (Supplementary Fig. 1)^17^. Conceivably, these differences could reflect variations in the specific odorants to which distinct musk-responsive subtypes are most sensitive^35^, and which may vary depending on the age and sex of mice contributing to the odor environment^38–41^. If so, we predicted that the age and/or sex of mice contributing to the odor environment at the time of EdU labeling might differentially affect the degree to which quantities of newborn OSNs of these subtypes are affected by olfactory deprivation. To test this, we compared open-side biases in newborn OSN quantities within UNO-treated juvenile males exposed to littermates alone versus those exposed to both littermates and adult parents (Fig. 4A, B). Strikingly, newborn Olfr1431 OSNs exhibited a 2-fold (*p* = 0.04) greater UNO effect size in the presence of adult mice compared to the absence (Fig. 4C, E), reflecting open-side biases of 4.8-fold (*p* = 0.009) and 2.5-fold (*p* = 0.009), respectively. By contrast, UNO effect sizes for newborn OSNs of subtypes Olfr235, Olfr1440, and Olfr1437, as well as the control subtype Olfr912, were not significantly affected by exposure to adults (*p* > 0.2; Supplementary Fig. 4, Fig. 4D, F). These findings are consistent with the hypothesis that stimulation-dependent increases in the quantities of newborn OSNs of musk-responsive subtypes can vary depending on the age of odor-emitting mice within the environment.

### Exposure to muscone intensifies deprivation-induced reductions in quantities of newborn OSNs of musk-responsive subtypes.

**Figure 5.**
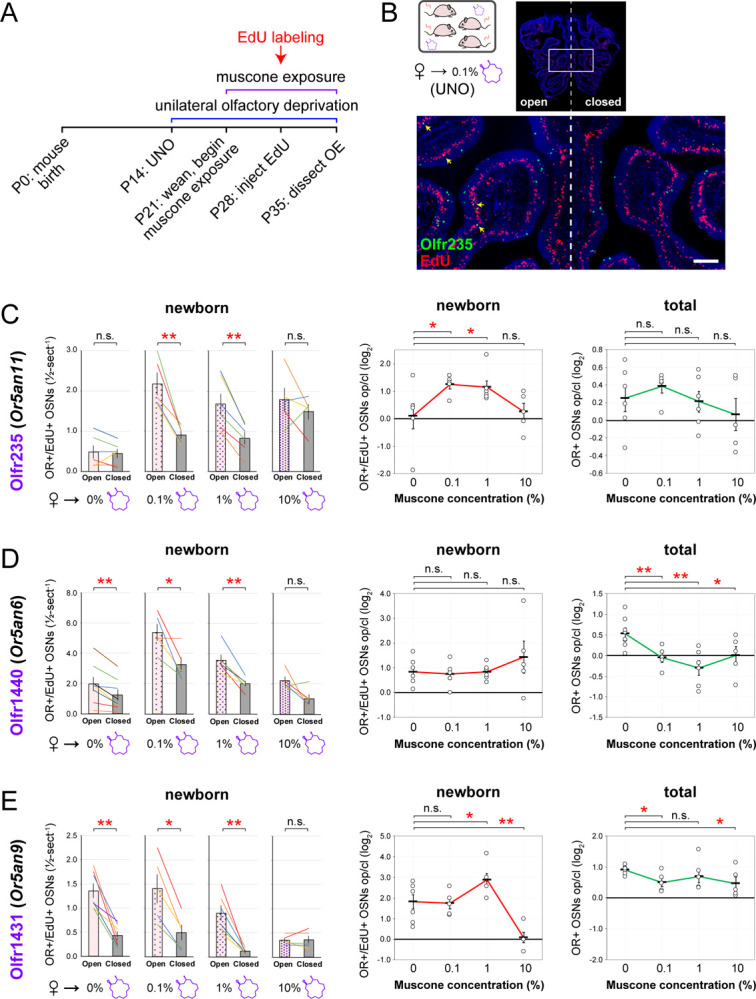
Exposure to muscone modulates deprivation-induced reductions in quantities of newborn OSNs of musk-responsive subtypes. A. Experimental timeline for the analysis of open-side biases in quantities of newborn OSNs of specific subtypes in female mice that were UNO treated at P14, weaned sex-separated at P21, exposed to varying concentrations of muscone starting at P21, EdU-labeled at P28, sacrificed at P35, and analyzed *via* OR-specific FISH and EdU staining. B. Representative image of an OE section stained for EdU and Olfr235 from a UNO-treated female mouse exposed to muscone (♀ → 0.1% muscone) at the time of EdU-labeling. OR+/EdU+ cells (*yellow arrows*) are newborn Olfr235 OSNs. Scale bar: 150 μm. C–E. Quantification of newborn OSNs (OR+/EdU+) (*left*) and UNO effect sizes for newborn (*middle*) and total (*right*) OSNs of subtypes Olfr235 (C), Olfr1440 (D), or Olfr1431 (E) within OEs of UNO-treated female mice exposed to 0, 0.1, 1, or 10% muscone at the time of EdU labeling. Musk-responsive subtypes are labeled in purple type. Each line or circle represents a distinct mouse (*n* = 5–10 mice per OSN subtype and condition). See also Supplementary Fig. 5.

Findings that the quantities of newborn OSNs of the musk-responsive subtypes Olfr235, Olfr1440, and Olfr1431 depend on olfactory stimulation presented an opportunity to investigate whether exposure to a cognate odorant for these subtypes (e.g., muscone) can drive changes in the quantities of newborn OSNs of specific subtypes. Considering that high levels of chronic odor stimulation can reduce OSN quantities^10,11,17^, presumably *via* reductions in OSN lifespan, we predicted the existence of a range of muscone concentrations sufficient to accelerate the birthrates of musk-responsive subtypes without adversely affecting their survival. To test this, we compared the effects of olfactory deprivation on quantities of newborn OSNs of musk responsive subtypes in female mice exposed to 0, 0.1, 1, or 10% muscone *via* a metal tea-ball containing a 1-mL aliquot of muscone solution deposited onto a piece of absorbent paper from weaning (P21) until dissection (P35) (Fig. 5A).

Interestingly, the effects of deprivation on quantities of newborn OSNs of subtype Olfr235 were found to depend strongly on the concentration of muscone to which mice were exposed, with biases of 1.1- (*p* = 0.8), 2.4- (*p* = 0.007), 2.0- (*p* = 0.002), and 1.2-fold (*p* = 0.4) observed for 0, 0.1, 1, and 10% muscone, respectively (Fig. 5B, C-*left*). Accordingly, the UNO effect size for quantities of newborn Olfr235 OSN was 13-fold higher in mice exposed to 0.1% muscone compared to 0% (*p* = 0.03), but not significantly increased compared to mice exposed to 10% (*p* = 0.4) (Fig. 5C-*middle*). Mean open-side biases in total Olfr235 OSN quantities were also affected by muscone concentration, peaking at 0.1%, and declining to a minimum at 10%, possibly reflecting reduced OSN survival in the presence of the higher muscone concentrations (Fig. 5C-*right*; Supplementary Fig. 5A).

In contrast to subtype Olfr235, open-side biases in quantities of newborn OSNs of subtypes Olfr1440 and Olfr1431 were more subtly affected by the concentration of muscone to which mice were exposed (Fig. 5D, E; Supplementary Fig. 5B, C), likely because both subtypes exhibit robust deprivation-induced biases even in the absence of muscone exposure. Most notably, subtype Olfr1431 exhibited a 1.6-fold greater UNO effect size for quantities of newborn OSNs in mice exposed to 1% muscone compared to 0% (*p* = 0.02), but a 20-fold smaller effect size in mice exposed to 10% (*p* = 0.002). For both subtypes, UNO effect sizes for quantities of total OSNs were reduced with increasing muscone concentration, indicating that muscone exposure exerts complex effects on the birth and survival of OSNs of these subtypes. As expected, muscone exposure did not significantly affect open-side biases in newborn or total OSN quantities of control subtypes Olfr912 and Olfr1463 (Supplementary Fig. 5D–G). Taken together, these findings indicate that the exposure of mice to muscone can potentiate deprivation-induced reductions in quantities of newborn OSNs of musk-responsive subtypes.

### Non-occluded females exposed to male odors or muscone exhibit elevated quantities of newborn OSNs of musk-responsive subtypes.

**Figure 6.**
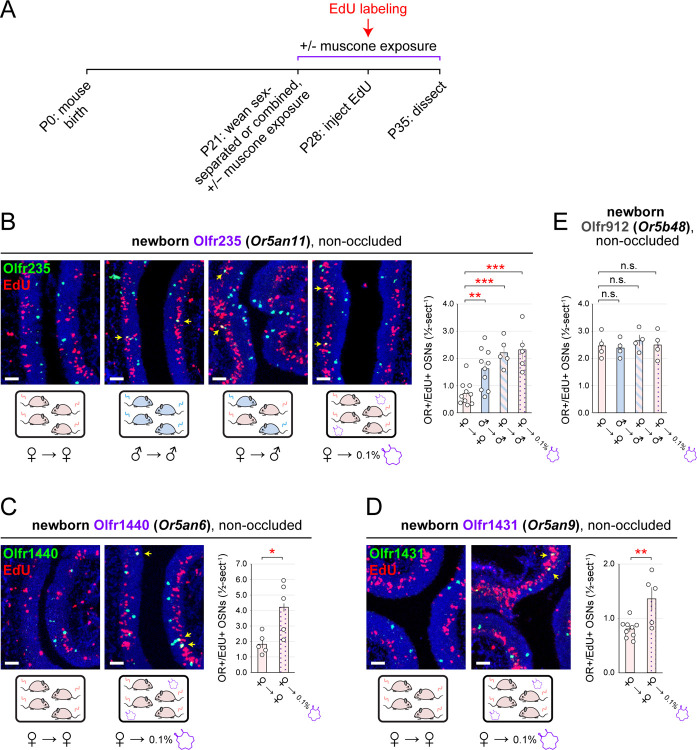
Exposure of non-occluded female mice to male odors or muscone induces elevated quantities of newborn OSNs of musk-responsive subtypes. A. Experimental timeline for the quantification of newborn OSNs of specific subtypes in non-occluded mice that were weaned sex-separated, sex-combined, or exposed to muscone starting at P21, EdU-labeled at P28, sacrificed at P35, and analyzed *via* OR-specific FISH and EdU staining. B. Representative images (*left*) of OE sections stained for Olfr235 mRNA and EdU, and quantification (*right*) of newborn (OR+/EdU+) Olfr235 OSNs within OEs from non-occluded females exposed to themselves (♀ → ♀), males exposed to themselves (♂ → ♂), females exposed to males (♀ → ♂), or females exposed to muscone (♀ → 0.1% muscone) at the time of EdU-labeling. C, D. Representative images (*left*) of OE sections stained for EdU and Olfr1440 (C) or Olfr1431 (D) mRNAs, and quantification (*right*) of newborn (OR+/EdU+) Olfr1440 (C) and Olfr1431 (D) OSNs within OEs of non-occluded females exposed to themselves (♀ → ♀) or to muscone (♀ → 0.1% muscone) at the time of EdU-labeling. E. Quantification of newborn (OR+/EdU+) Olfr912 OSNs within OEs from non-occluded females exposed to themselves (♀ → ♀), males exposed to themselves (♂ → ♂), females exposed to males (♀ → ♂), or females exposed to muscone (♀ → 0.1% muscone) at the time of EdU-labeling. OR+/EdU+ cells (*yellow arrows*) are newborn OSNs of the indicated subtype. Scale bars: 50 μm. Musk-responsive and control subtypes are labeled in purple and gray type, respectively. Each circle represents a distinct mouse (*n* = 4–10 mice per OSN subtype and condition).

Findings that exposure to male odors or muscone enhances deprivation-induced reductions in quantities of newborn OSNs of musk-responsive subtypes are consistent with the hypothesis that musk-like odors can increase the quantity of newborn OSNs of these subtypes. However, because these observations were based on the use of UNO, a procedure that causes physiological and sensory changes beyond odor deprivation^31^, we sought to test this hypothesis using a more direct approach. To this end, we assessed the effects of exposing non-occluded mice to male odors or 0.1% muscone on quantities of newborn OSNs of musk-responsive subtypes (Fig. 6A). Strikingly, subtype Olfr235 exhibited significantly greater quantities of newborn OSNs in mice exposed to male odors or muscone [2.2-fold (*p* = 0.004) in sex-separated males, 3.1-fold (*p* = 0.00006) in sex-combined females, and 3.2-fold (*p* = 0.0002) in muscone-exposed females] compared to mice isolated from these odors (sex-separated females) (Fig 6B). Similarly, analyses of subtypes Olfr1440 and Olfr1431 revealed 2.3-fold (*p* = 0.02), and 1.6-fold (*p* = 0.003) greater quantities of newborn OSNs in muscone-exposed compared to unexposed females (Fig. 6C, D). As expected, the quantities of newborn OSNs of the negative control subtype Olfr912 did not significantly differ between treatment groups (*p* > 0.6) (Fig. 6E). Taken together, these findings further support the hypothesis that the exposure of mice to male or musk odors can increase the quantities of newborn OSNs of musk-responsive subtypes.

### Stimulation-dependent changes in newborn OSN quantities are observed immediately after neurogenesis, consistent with a mechanism involving altered birthrate.

**Figure 7.**
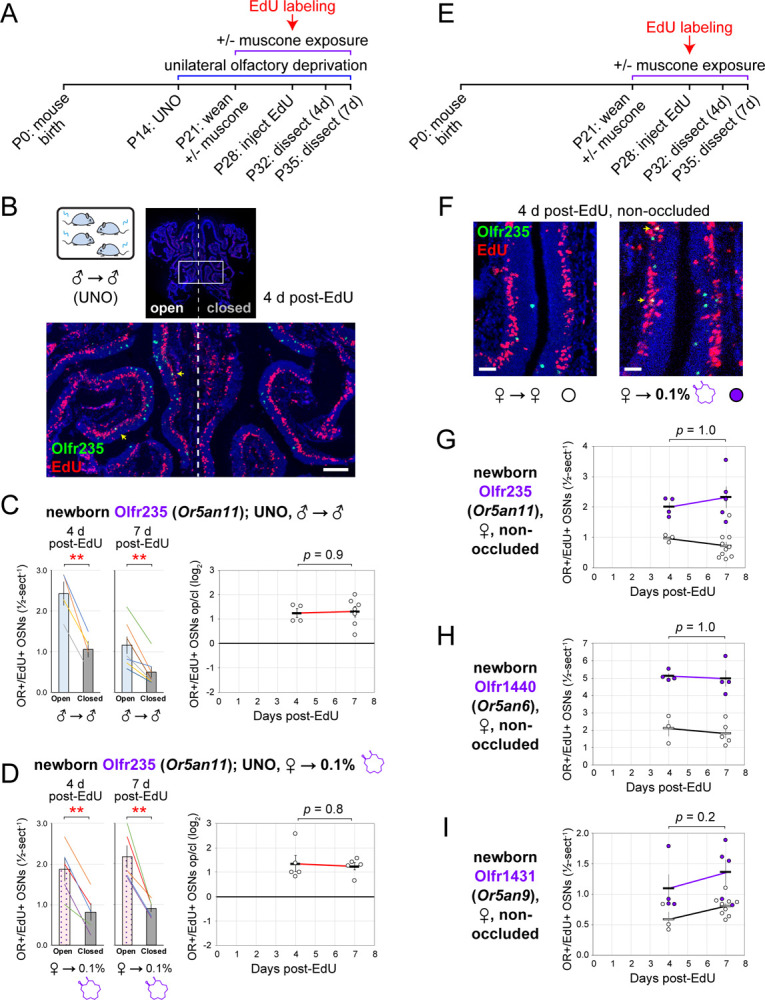
Stimulation-dependent changes in newborn OSN quantities are observed immediately after neurogenesis and stable thereafter, consistent with a mechanism involving altered birthrate. A. Experimental timeline for analysis of the time-dependence of open-side biases in quantities of newborn OSNs of specific subtypes in mice that were UNO treated at P14, weaned sex-separated at P21, exposed to muscone (subset of mice) starting at P21, EdU-labeled at P28, sacrificed at P32 (4 d post-EdU) or P35 (7 d post-EdU), and analyzed *via* OR-specific FISH and EdU staining. B. Representative image of an OE section stained for EdU and Olfr235 from UNO-treated male mice exposed to themselves (♂ → ♂) at the time of EdU-labeling and sacrificed 4 d post-EdU. OR+/EdU+ cells (*yellow arrows*) are newborn Olfr235 OSNs. Scale bar: 150 μm. C, D. Quantification of (*left*) and UNO effect sizes for (*right*) newborn Olfr235 OSNs (OR+/EdU+) within OEs of UNO-treated males exposed to themselves (♂ → ♂) (C) or females exposed to muscone (♀ → 0.1% muscone) (D) at the time of EdU labeling. Each line or circle represents a distinct mouse (*n* = 4–7 mice per OSN subtype and condition). E. Experimental timeline for analysis of the time-dependence of muscone exposure-induced increases in quantities of newborn OSNs of specific subtypes non-occluded mice that were weaned sex-separated at P21, exposed to muscone (subset of mice) starting at P21, EdU-labeled at P28, sacrificed at P32 (4 d post-EdU) or P35 (7 d post-EdU), and analyzed *via* OR-specific FISH and EdU staining. F. Representative images of OE sections stained for Olfr235 mRNA and EdU, from non-occluded females exposed to themselves (♀ → ♀; *left*) or to muscone (♀ → 0.1% muscone; *right*) at the time of EdU-labeling. Scale bars: 50 μm. G–I. Quantification of newborn (OR+/EdU+) Olfr235 (G), Olfr1440 (H), and Olfr1431 (I) OSNs within OEs of non-occluded females exposed to themselves (♀ → ♀; *black circles*) or to muscone (♀ → 0.1% muscone; *purple circles*) at the time of EdU-labeling. Musk-responsive subtypes are labeled in purple type. Each circle represents a distinct mouse (*n* = 4–10 mice per OSN subtype and condition). See also Supplementary Fig. 6.

Stimulation-dependent increases in the quantities of newborn OSNs of musk-responsive subtypes could, in theory, be caused by a mechanism that selectively accelerates the rates with which these subtypes are generated or, alternatively, the rates with which they are selectively enriched following their generation (e.g., *via* enhanced survival or OR switching^42^). If differences in newborn OSN quantities are mediated by selective enrichment, we would expect them to become more pronounced over time following neurogenesis, as a subset of newborn OSNs exhibit enhanced survival or switch their OR identity in the presence of stimulation. By contrast, if changes are mediated by accelerated birthrates of specific OSN subtypes, increases in newborn OSN quantities should appear immediately upon neurogenesis and remain stable over time. To distinguish between these potential mechanisms, we compared stimulation-dependent changes in quantities of newborn musk-responsive OSNs at two timepoints: 4 days post-EdU, the earliest stage during OSN differentiation when OR transcripts can be consistently detected *via* FISH^30,43^, and three days later (7 days post-EdU) (Fig. 7A, E). In initial experiments, deprivation-induced reductions in the quantities of newborn OSNs of musk-responsive subtypes were analyzed in sex-separated male, female, and 0.1% muscone-exposed female mice (Fig. 7A). In UNO-treated male mice, open-side biases of near-equivalent magnitude were observed for quantities of newborn OSNs at both 4 and 7 days for musk-responsive subtypes Olfr235 [2.3-fold (*p* = 0.003) and 2.3-fold (*p* = 0.005), respectively], Olfr1440 [1.8-fold (*p* = 0.03) and 1.4-fold (*p* = 0.04), respectively] and Olfr1431 [2.5-fold (*p* = 0.009) and 2.4-fold (*p* = 0.0009), respectively]. Accordingly, no significant differences in UNO effects sizes were observed between the two timepoints for any of the three subtypes (*p* > 0.7) (Fig. 7B, C; Supplementary Fig. 6A, B). Likewise, muscone-exposed, UNO-treated females exhibited highly similar open-side biases in newborn Olfr235 OSN quantities at 4 and 7 days post-EdU [2.3-fold (*p* = 0.002) and 2.4-fold (*p* = 0.007), respectively], and corresponding UNO effect sizes that did not differ significantly between the timepoints (*p* = 0.8) (Fig. 7D; Supplementary Fig. 6C). As expected, no significant open-side biases in newborn OSN quantities at either of the two timepoints, or differences in corresponding UNO effect sizes, were observed for control subtypes Olfr912 or Olfr1463 in UNO-treated male (*p* = 0.8) (Supplementary Fig. 6D, E) or female mice exposed to muscone (*p* = 0.4) (Supplementary Fig. 6F), or for subtype Olfr235 in unexposed females (*p* = 0.9) (Supplementary Fig. 6G).

We next assessed in non-occluded female mice the stability of muscone-dependent increases in quantities of newborn musk-responsive OSNs between 4 and 7 days post-EdU (Fig. 7E). As was observed for UNO-treatment, non-occluded mice exposed to muscone exhibited statistically indistinguishable increases in quantities of newborn OSNs between the two timepoints for subtypes Olfr235 (*p* = 1.0), Olfr1440 (*p* = 1.0), and Olfr1431 (*p* = 0.2) (Fig. 7F–I), as well as the control subtype Olfr912 (*p* = 0.4) (Supplementary Fig. 6H). Taken together, these results support the hypothesis that olfactory stimulation-dependent increases in the quantities of newborn OSNs of musk responsive subtypes reflect subtype-selective changes in OSN birthrate.

### Muscone exposure-dependent increases in quantities of newborn OSNs of musk-responsive subtypes persist into adulthood.

Findings that the exposure of juvenile mice to male or musk-like odors increases quantities of newborn OSNs of subtypes responsive to these odors raise the question of whether this phenomenon is limited to early life or, rather, persists into adulthood. To begin to address this question, we compared newborn OSN quantities in 9-week-old non-occluded female mice that were either exposed or unexposed to muscone following weaning (P21), and EdU-treated at ~8 weeks of age (P56-P58) (Supplementary Fig. 7A). Strikingly, compared to unexposed controls, muscone-exposed adults exhibited robust increases in quantities of newborn OSNs of musk-responsive subtypes: 1.5-fold (*p* = 0.06) for subtype Olfr235, 2.2-fold (*p* = 0.02) for Olfr1440, and 1.9-fold (*p* = 0.04) for Olfr1431, although increases for subtype Olfr235 did not reach the statistical significance threshold of 0.05 (Supplementary Fig. 7B–D). As expected, exposure to muscone did not cause a significant increase in newborn OSNs of control subtype Olfr912 (1.05-fold; *p* = 0.4) (Supplementary Fig. 7E). These findings reveal that the capacity for muscone-induced increases in quantities of newborn musk-responsive OSNs is not limited to the juvenile stage.

## Discussion

### The birthrates of some musk/male-odor-responsive OSN subtypes are accelerated by exposure to those odors.

It is well established that UNO reduces the rate of neurogenesis on the closed side of the OE relative to the open^44–47^. Recently, these deprivation-induced reductions were found to reflect diminished birthrates of only a fraction of the ~1200 OSN subtypes, suggesting that unknown olfactory stimuli sustain the birthrates of these subtypes^30^. Here we have described experiments to elucidate the nature of the stimuli that promote the birthrates of specific OSN subtypes, and presented evidence that these stimuli include discrete odorants that selectively stimulate the same subtypes whose birthrates are accelerated. Our study took advantage of previous findings that a group of closely related musk-responsive OSN subtypes are more highly represented in the OEs of mice exposed to male odors compared to mice isolated from them, and that some of these subtypes are responsive to male-specific odors^17^ (Supplementary Fig. 1). These data suggested that one or more components of male odors and, potentially, musk-like odors, might accelerate the birthrates of these OSN subtypes. Here, using both scRNA-seq-based and histological approaches, we have found that, indeed, olfactory deprivation in juvenile males reduces quantities of newborn OSNs of musk-responsive subtypes (Figs. 1, 2). We further found that exposure to male odors and/or muscone: 1) intensifies deprivation-induced reductions in quantities of newborn Olfr235 OSNs in female mice (Fig. 3, 5), and 2) increases quantities of newborn Olfr235, Olfr1440, and Olfr1431 OSNs within the OEs of non-occluded females (Fig. 6). These findings support the hypothesis that the stimuli that regulate the birthrates of specific subtypes are discrete cognate odors for those subtypes.

Findings that quantities of newborn OSNs of the musk-responsive subtype Olfr235 are increased in the presence of male mice suggest the possibility that one or more musk-like molecules to which Olfr235 OSNs selectively respond may be emitted by male but not female mice. This would explain previous findings that total Olfr235 OSNs are more highly represented in male mice and females housed with males compared to sex-separated females^17,32^. Curiously, two other musk-responsive subtypes that likewise displayed a higher representation in males and females housed with males, Olfr1440 and Olfr1431^17^, showed stimulation-dependent changes in newborn OSN quantities in both male and female mice (Fig. 3), while a third such subtype, Olfr1437, exhibited no stimulation-dependent changes in newborn OSN quantities in mice of either sex (Fig. 2 and not shown). Considering the close relationship of the ORs that define musk-responsive OSN subtypes^34^, the mechanism underlying these differences is intriguing. One hypothetical explanation is that musk-responsive OSN subtypes vary in their sensitivity to distinct musk-like odorant molecules^35,48^, which may be differentially emitted by mice in an age- and sex-dependent manner. Indeed, mouse odor profiles are known to vary considerably as a function of age and sex^38–41^. In support of this hypothesis, we found that exposure of juvenile mice to adults intensifies deprivation-induced reductions in quantities of newborn Olfr1431 OSNs (Figure 4). Future identification of the natural odorants to which these subtypes respond will be important to enable further testing of this hypothesis.

### Stimulation-dependent changes in quantities of newborn OSNs of male/musk-responsive subtypes reflect altered rates of neurogenesis.

A previous study found evidence that reduced quantities of newborn OSNs of specific subtypes observed following naris occlusion are most consistent with a mechanism involving decreased OSN birthrates, not increased rates of newborn OSN apoptosis or subtype switching^30^. This evidence included findings that UNO-induced open-side biases in the quantities of newborn OSNs of specific subtypes display a near-maximum shortly following the onset of OR expression (4 days post-EdU) and do not differ significantly thereafter. These results appear inconsistent with a mechanism involving the selective apoptosis or subtype switching of newborn OSNs, which would be expected to cause a gradual increase in open-side biases over time. Similarly, the present study finds that UNO-induced open-side biases in quantities of newborn OSNs of musk/male odor-responsive subtypes do not differ significantly between 4 and 7 days post-EdU (Fig. 7B–D). Moreover, findings from the present study based on the exposure of non-occluded mice to male or musk odors (Fig. 6) indicate that altered quantities of newborn OSNs of musk-responsive subtypes cannot be explained by hypothetical UNO-induced increases in the rates of newborn OSN death or subtype switching. Observations that the magnitude of muscone exposure-induced increases in newborn OSN quantities do not differ significantly between 4 and 7 days post-EdU (Fig. 7F–I) reinforce that these changes are unlikely due to apoptosis or OR switching, although they do not strictly exclude the possibility that such mechanisms occur prior to 4 days post-EdU. Taken together, these results provide strong evidence that male/musk odor-dependent changes in the quantities of newborn OSNs reflect altered OSN birthrates.

### How do discrete odors accelerate the birthrates of specific OSN subtypes?

Our findings that specific OSN subtypes exhibit accelerated birthrates following the exposure of mice to cognate odors for these subtypes indicate that this process occurs *via* a mechanism that is highly selective with respect to the stimulating odors and the subtypes whose birthrates are accelerated. Considering that horizontal basal cells (HBCs) and globose basal cells (GBCs), the stem and progenitor cells that give rise to new OSNs, lack ORs and signal transduction molecules needed to detect and respond to odors, we hypothesize the existence of a signaling pathway from mature OSNs to HBCs or GBCs that alters the rates at which OSNs of specific subtypes are born. Findings from the present study and a previous one^30^ indicate that this signaling capacity may be limited to only a fraction of OSN subtypes, since a majority of subtypes do not exhibit accelerated neurogenesis upon stimulation^30^. Subtype Olfr912, for example, which detects the male-specific odor component 2-sec-butyl-4,5-dihydrothiazole (SBT)^32^, exhibits no increase in birthrate upon exposure of female mice to male odors, and was therefore employed in this study as a control subtype (Figs. 2–4, 6). We hypothesize that the receipt of odor-derived signals by HBCs or GBCs alters OR choices or amplifies choices that have already been made. Elucidating the nature of odor-dependent signals received by HBCs/GBCs, as well as the mechanism by which these signals accelerate the birthrates of specific OSN subtypes are important areas of future investigation.

### What function does odor stimulation-dependent neurogenesis serve?

**Figure 8.**
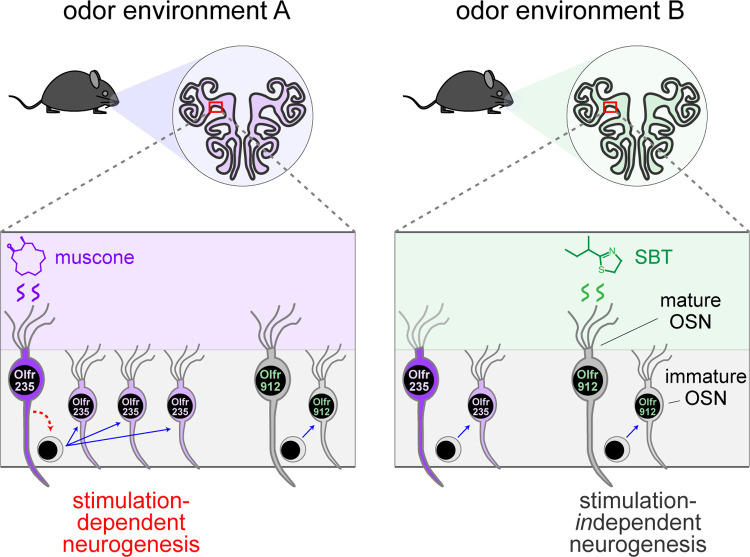
Model for the selective acceleration of the birthrates of specific OSN subtypes by discrete odors that stimulate them. A fraction of OSN subtypes (e.g., Olfr235), upon stimulation by discrete odors (e.g., muscone), undergo accelerated rates of neurogenesis. Other subtypes (e.g., Olfr912) do not exhibit altered rates of neurogenesis upon stimulation by discrete odors that stimulate them (e.g., SBT). A hypothetical mechanism involves selective stimulation-dependent signaling by mature OSNs of specific subtypes to neural progenitors.

Because OSN differentiation entails the stochastic process of singular OR choice^8,9^, it has long been assumed that OSN neurogenesis is entirely stochastic with respect to OR identity. Thus, unlike other regions of the nervous system where persistent neurogenesis is known to play important adaptive roles^5–7^, life-long neurogenesis within the OE is generally assumed to serve the merely homeostatic function of replacing neurons lost to turnover and injury^4^. Results of the present study, together with those of a previous one^30^, challenge these assumptions by demonstrating that neurogenesis is not entirely stochastic with respect to subtype, but rather that the birthrates of a fraction of OSN subtypes can be selectively and directionally regulated by discrete odor experiences (Fig. 8). These findings suggest the possibility that persistent neurogenesis within the OE may serve an unknown adaptive function in addition to the known homeostatic one. It is conceivable, for example, that the acceleration of the birthrates of specific OSN subtypes could selectively enhance sensitivity to odors detected by those subtypes by increasing their representation within the OE^49–52^. Under this scenario, OSNs of affected subtypes might have baseline (unamplified) representations that lie within the dynamic range for signaling to projection neurons under physiological concentrations of cognate odors, such that amplification *via* neurogenesis could enhance their sensitivity. This effect could have relevance to observations in both rodents and humans that exposure to specific odors can dramatically increase sensitivity to them^22–27^. Alternatively, or in addition, OSNs produced *via* odor-dependent neurogenesis could conceivably enable the formation of new OB glomeruli and connections with projection neurons^53–56^. Under this scenario, stimulation-dependent neurogenesis of specific subtypes could alter inputs to the olfactory cortex and thereby regulate the perception of, and behavioral responses to, specific odors.

Results of the present study demonstrate that the birthrates of musk-responsive subtypes can be regulated by exposure to musk odors, a group of compounds that are naturally emitted by numerous mammalian species^57–59^ and, in mice, activate a small number of related and evolutionarily conserved odorant receptors^34,35,60^. Findings that odors emitted by mice can also activate these subtypes^17,32^ suggest that musk-like compounds may contribute to the odors emitted by mice. For some mammals, musk odors are known to function in attracting mates, marking territory, and deterring predators^61,62^. Moreover, exposure to musk odors has been reported to cause physiological changes in some mammals, including humans, suggesting that they can function as semiochemicals^63,64^. In mice, the physiological functions of musk odors, if any, are unknown, although they have been found to be selectively attractive to male mice^48^. Results from the present study suggest that exposure to musk compounds may mediate adaptive changes within the mouse OE that reflect a special salience for these odors.

## Methods

### Experimental model and subject details

All procedures involving mice were carried out in accordance with NIH standards and approved by the University of Colorado Anschutz Medical Campus Institutional Animal Care and Use Committee (IACUC). For all experiments described, tissue samples were obtained from male and female C57Bl/6J mice age P35 or P65 at the time of sacrifice. Except for adult-exposed mice, which remained with their parents until sacrifice, all mice were weaned at P21 and group-housed either sex-separated or sex-combined in standard cages at a density of no more than 5 mice/cage. Additional details about animals used in this study can be found in Supplementary Table 1.

### Method details

- Unilateral naris occlusion (UNO). P14 pups were anesthetized using isoflurane (completeness of anesthesia confirmed through a tail pinch), and then immediately subjected to electrocautery for ~5 seconds on the right nostril under a dissecting microscope. During electrocautery care was taken to avoid contact of the electrocautery unit with any non-superficial tissues. Pups were examined daily following the procedure to ensure complete blockage of the right nostril through scar formation (typically ~3–5 days after the procedure) and normal development and activity.
- Muscone exposure. A 1-mL aliquot of muscone (Ambeed, Inc.; A275816) solution (0.1, 1, or 10% in propylene glycol) was applied to a compactly folded piece of absorbent paper (KimTech), which was placed in a metal tea all that was suspended in a standard ventilated mouse cage. Mice were exposed to muscone from weaning (P21) until sacrifice (P35 or P65), with the muscone odorant changed every other day.
- 2-Deoxy-5-ethynyluridine (EdU) injections. EdU (Carbosynth; NE08701) was administered to C57Bl/6J mice at P28 or P56–58 (two intraperitoneal injections/day of 10 mg/mL EdU in sterile PBS; 50 mg/kg mouse body weight/injection) spaced three hours apart. For additional details, see^37^.
- *In situ* hybridization (ISH) probe design and production. ISH probes were designed to span 500–1000 base pairs and were targeted to CDS and/or UTR regions of each mRNA (see Supplementary Table 2). Probes were designed to minimize cross-hybridization with off-target mRNAs, which was assessed using BLAST. For the detection of specific ORs, probes targeting multiple gene regions were typically generated and tested. Probe sequences were amplified by PCR using specific primers (Table S2), inserted into the pCRII-TOPO vector (ThermoFisher), and confirmed by restriction analysis and sequencing. DIG- and FITC-labeled antisense RNA probes were generated from 1 μg of linearized plasmid template using T7 or Sp6 RNA polymerases (NEB) and DIG-11-UTP (Roche), treated with DNaseI (Promega), ethanol precipitated, and dissolved in a 30-μL volume of water. For additional details, see^37^. Additional details about the preparation of probes used in this study can be found in Supplementary Table 2.
- One-color RNA fluorescent *in situ* hybridization (RNA-FISH) combined with EdU staining *via* click chemistry. OEs were dissected from experimental mice (age P35 or P65), placed in a cryomold containing OCT, flash-frozen in liquid-nitrogen-cooled isopentane, and stored at −80 °C until sectioning. Tissues were cut into 12-μm thick cryo-sections, placed onto slides, and stored at −80 °C until staining. Slide-mounted sections were warmed (37 °C, 5 min), equilibrated in phosphate-buffered saline (PBS; pH 7.2; 3 min, room temperature [RT]), fixed in paraformaldehyde (PFA; 4% in PBS; 10 min, RT), washed in PBS (3 min, RT), permeabilized with Triton-X-100 (0.5% in PBS; 10 min, RT) followed by sodium dodecyl sulfate (1% in PBS; 3 min, RT), washed in PBS (3 × 3 min, RT), incubated in acetylation solution (triethanolamine [0.1 M; pH 7.5], acetic anhydride [0.25%]; 10 min, RT), washed in PBS (3 × 3 min, RT), incubated in hybridization solution (formamide [50%], SSC [5×], Denhardts [5×], yeast tRNA [250 μg/mL], herring sperm DNA [500 μg/mL], heparin [50 μg/mL], EDTA [2.5 mM], Tween-20 [0.1%], CHAPS [0.25 %]; 30 min, RT), hybridized with a DIG-labeled antisense RNA probe (1:750 in hybridization solution; 16 hr, 65 °C), washed with SSC (5×; 1 × 5 min, 65 °C), washed with SSC (0.2×; 4 × 20 min, 65 °C), incubated in H_2_O_2_ (3% in TN [Tris-HCl (0.1 M; pH 7.5), 0.15 M NaCl]; 30 min, RT), washed in TNT (Tween-20 [0.05%] in TN; 5 × 3 min, RT), incubated in TNB (Blocking Reagent [Perkin Elmer; 0.05% in TN]; 30 min, RT), incubated with anti-DIG-POD antibody (Roche; 1:1000 in TNB; 12 hr, 4 °C), and washed in TNT (3 × 20 min, RT). Fluorescent signals corresponding to the target transcript were generated using the Tyramide Signal Amplification (TSA) Plus Fluorescein Kit (Perkin Elmer) according to the manufacturer’s instructions. Slides were washed in 3% BSA in PBS (2 × 5 min, RT, with gentle rocking), incubated with EdU reaction solution (4 mM CuSO_4_, 4 μM Sulfo-Cyanine 3 Azide [Lumiprobe], 100 mM sodium ascorbate [prepared fresh], in PBS; 30 min, RT, in darkness), and washed with 3% BSA in PBS (2 × 3 min, RT). Slides were washed in TNT (2 × 3 min, RT), incubated in DAPI (300 nM in TN; 3 min, RT), washed in TNT (1 × 3 min, RT), and mounted using Vectashield (Vector Laboratories). For additional details, see^37^.
- Image acquisition and processing. Images were acquired using a Zeiss LSM 900 with Airyscan 2 microscope with an automated stage and Zen Blue software (Zeiss). Mosaic images were stitched, and each fluorescence channel was adjusted individually to enhance contrast and reduce background using Zen Blue software. Images were exported in jpg format, rotated and cropped using Adobe Photoshop, and labeled using Adobe Illustrator (Adobe Systems). For additional details, see^37^.
- Quality criteria for sectioned OEs. For each mouse, UNO efficiency was determined by staining OE sections for *S100a5* mRNA *via* one-color RNA-FISH. For each section analyzed, *S100a5* mRNA intensities were evaluated within paired regions on the two sides of each OE section. OEs from UNO-treated mice were excluded from further analysis if the mean *S100a5* mRNA staining intensity on the open side of the OE was not clearly greater than that on the closed side. All sections were also assessed for left-right symmetry and for intactness. Individual OE sections were excluded if they were not visually symmetrical or were less than 90% intact. No data were otherwise excluded. For additional details, see^37^.

### Quantification and statistical analysis

- scRNA-seq-based analysis of UNO-induced changes in subtype-specific OSN quantities. OSNs were quantified within scRNA-seq datasets generated from the open and closed sides of the OE of a male mouse that had been UNO-treated at P14 and euthanized at P28^30^ (https://www.ncbi.nlm.nih.gov/geo/query/acc.cgi?acc=GSE157119). OSNs of interest were quantified from the open and closed datasets using the Loupe Cell Browser (10X Genomics). Newborn OSNs of specific subtypes were identified by expression of *Gap43* (Log_2_ UMI > 1) and a specific OR gene (Log_2_ UMI > 3). Total OSNs of specific subtypes were identified by expression of *Gap43* (Log_2_ UMI > 1) and/or *Omp* (Log_2_ UMI > 3) and a specific OR gene (Log_2_ UMI > 3). The percentage of the total cell population represented by newborn and total OSNs of specific subtypes were determined from the total number of cells in each dataset.
- Quantification of OR+ and OR+/EdU+ cellular abundance. Cell counts corresponding to each mouse were determined from images of a series of at least 5 stained coronal sections located ~400 μm apart and spanning the anterior-posterior length of the OE. Counting was performed separately on the right and left side of each OE section, with the experimenter blinded to sample groups and section orientations. The open and closed sides of OEs from UNO-treated mice were determined after counting was complete using fluorescent signals corresponding to *S100a5* mRNA (on adjacent sections). Cells containing EdU+ nuclei (Cy3-signal) that were at least 50% overlapping with OR mRNA signals (FITC-signal) were considered EdU+/OR+ OSNs. For additional details, see^37^.
- Statistics. For all statistical analyses, a significance threshold of *p* < 0.05 was used. Statistical analyses of comparisons of OSN counts between the open and closed sides were performed using a two-tailed paired *t*-test, in which the two sides of an OE were paired. This enabled statistical analyses of differences between the two sides independent of OSN number and staining variance between sections. For comparisons of samples between different non-occluded animals, a two-tailed unpaired *t*-test was used. For comparisons of UNO effect sizes between 4 and 7 days post-EdU, a two-tailed unpaired *t*-test was used. For comparisons of differences in quantities of newborn OSNs of musk-responsive subtypes at 4 and 7 days post-EdU between non-occluded mice exposed and unexposed to muscone, a two sample ANOVA - fixed-test, using F distribution (right-tailed) was used. Data presented in figures represent mean +/− SEM. For additional details, see^37^.
- Sample-size estimation. Results from previous studies^17,30^ were used to determine an appropriate sample size for comparing the number of OR+ and OR+/EdU+ OSNs on the open and closed sides of the OE. Previously, it was found that for an OR with a typical expression frequency (~0.1%) and an effect size of ~2-fold, 12 OE sections taken from four different animals were sufficient to find a highly statistically significant difference (*p* < 0.001; two-tailed paired *t* test). In the current study, the sample sizes used were typically larger than this. For comparisons between OE sections from different animals, results from previous analyses^17,30^ were again used to determine an appropriate sample size. Previously, we had found that for an OR with a typical expression frequency (~0.1%) and an effect size of ~2-fold, 20 OE sections taken from four different animals was sufficient to find a highly statistically significant difference between different animals (*p* < 0.01; two-tailed unpaired *t* test). For additional details, see^37^.

## Supplementary Material

1
