# Supplementary material for: In mice, discrete odors can selectively promote the neurogenesis of sensory neuron subtypes that they stimulate
Source: bioRxiv. 2024 Feb 12:2024.02.10.579748. Preprint. [Version 1] doi: 10.1101/2024.02.10.579748 (PMC10888860; doi:10.1101/2024.02.10.579748)
Supplement: Supplementary file 1 [file NIHPP2024.02.10.579748V1-supplement-1.pdf]

bioRxiv preprint doi: https://doi.org/10.1101/2024.02.10.579748; this version posted February 12, 2024. The copyright holder for this preprint (which was not certified by peer review) is the author/funder, who has granted bioRxiv a license to display the preprint in perpetuity. It is made available under aCC-BY 4.0 International license.

Supplementary Figure 1

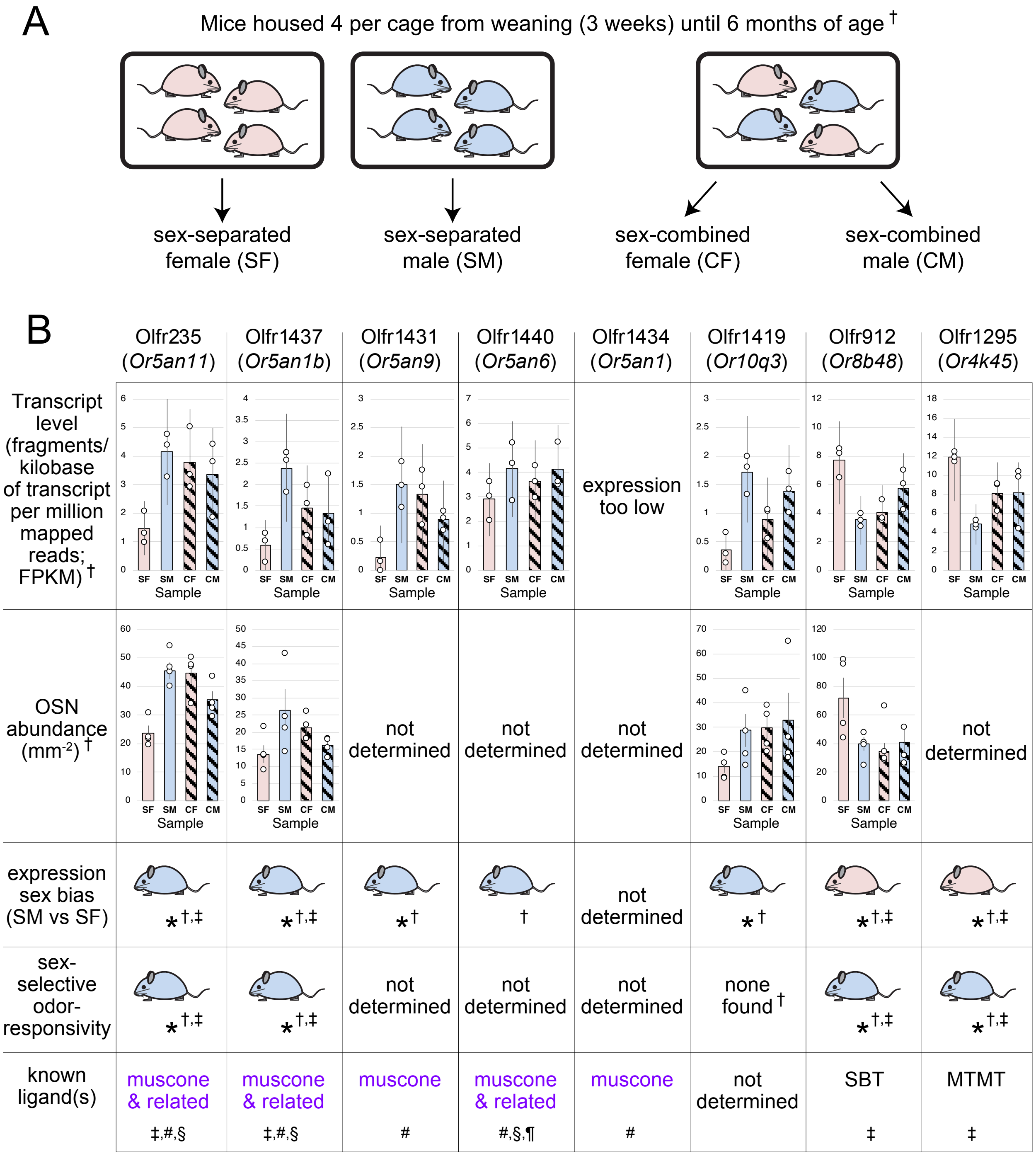

bioRxiv preprint doi: https://doi.org/10.1101/2024.02.10.579748; this version posted February 12, 2024. The copyright holder for this preprint (which was not certified by peer review) is the author/funder, who has granted bioRxiv a license to display the preprint in perpetuity. It is made available under aCC-BY 4.0 International license.

Supplementary Figure 1. Identification of OSN subtypes that are candidates for undergoing sex-specific- and/or musk odor-accelerated neurogenesis. A. Experimental schematic for the identification of OSN subtypes whose representation depends on exposure to sex-specific odors. Datasets used in this study were generated from mice that were housed either sex-separated or sex-combined from weaning (P21) until the time of sacrifice (6 months), followed by OEs dissection and analysis *via* bulk RNA-seq [17,65]. B. OSN subtypes previously identified as responsive to sex-specific odors and/or musk-like odors. SBT, 2-sec-butyl-4,5-dihydrothiazole; MTMT, (methylthio)methanethiol; †, [17,65]; ‡, [32]; #, [34]; §, [35]; ¶, [60].

bioRxiv preprint doi: https://doi.org/10.1101/2024.02.10.579748; this version posted February 12, 2024. The copyright holder for this preprint (which was not certified by peer review) is the author/funder, who has granted bioRxiv a license to display the preprint in perpetuity. It is made available under aCC-BY 4.0 International license.

Supplementary Figure 2

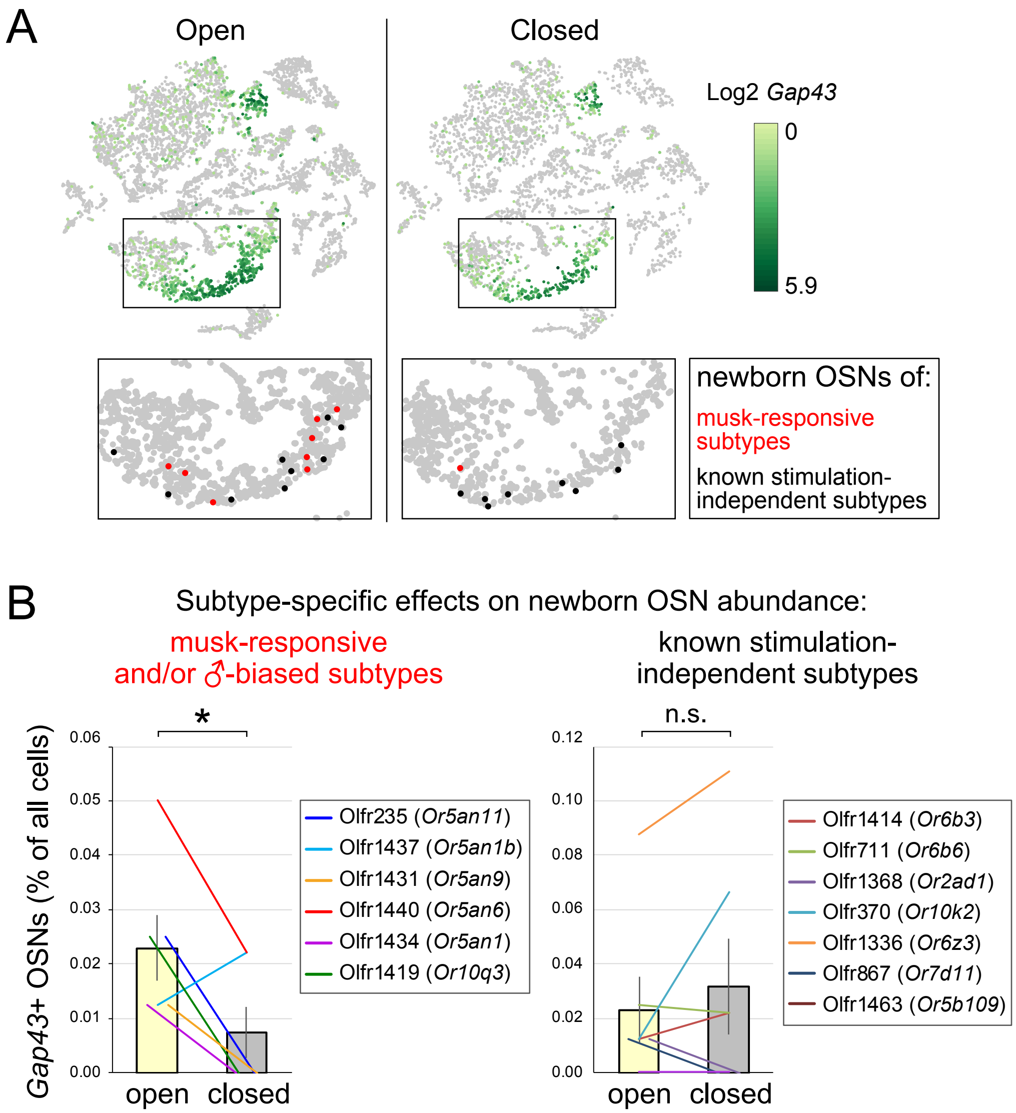

Supplementary Figure 2. scRNA-seq analysis of OEs from UNO-treated male mice shows reduced quantities of newborn OSNs of musk-responsive and/or male-biased subtypes on the closed side of the OE relative to the open. A. (*top*) *t*-SNE plot representation of scRNA-seq datasets corresponding to the open (*left*) and closed (*right*) sides of the OE, showing *Gap43* (immature OSNs) expression [30]. (*bottom*) Identification of immature OSNs of the 5 known musk-responsive subtypes (Olfr235, Olfr1440, Olfr1431, Olfr1434, Olfr1437) within the open and closed datasets. B. Quantification of individual (lines) and average (bars) percentages of the OE cell population represented by immature (*Gap43+*) OSNs of musk-responsive and/or male-biased subtypes (*left*) or known stimulation-*in*dependent subtypes (*right*) within the open and closed datasets. Related to Fig. 1.

bioRxiv preprint doi: https://doi.org/10.1101/2024.02.10.579748; this version posted February 12, 2024. The copyright holder for this preprint (which was not certified by peer review) is the author/funder, who has granted bioRxiv a license to display the preprint in perpetuity. It is made available under aCC-BY 4.0 International license.

Supplementary Figure 3

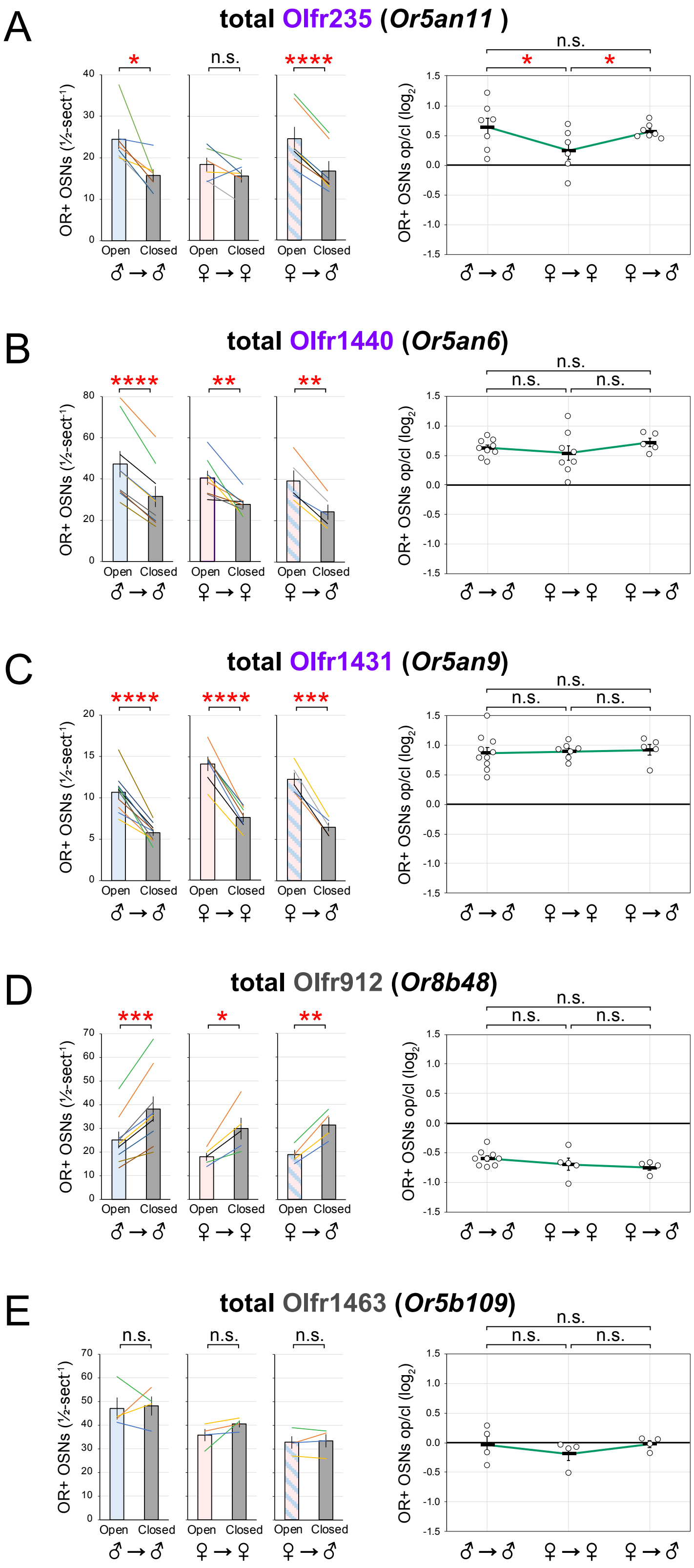

bioRxiv preprint doi: https://doi.org/10.1101/2024.02.10.579748; this version posted February 12, 2024. The copyright holder for this preprint (which was not certified by peer review) is the author/funder, who has granted bioRxiv a license to display the preprint in perpetuity. It is made available under aCC-BY 4.0 International license.

Supplementary Figure 3. Olfactory deprivation-induced reductions in quantities of newborn Olfr235 OSNs depend on exposure to male odors. A–E. Quantification (*left*) and UNO effect sizes (*right*) of total OSNs (OR+) of subtypes Olfr235 (A), Olfr1440 (B), Olfr1431 (C), Olfr912 (D), and Olfr1463 (E) within OEs of UNO-treated male mice exposed to themselves (♂ → ♂), female mice exposed to themselves (♀ → ♀), or female mice exposed to male mice (♀ → ♂) at the time of EdU labeling. Musk-responsive and control subtypes are labeled in purple and gray type, respectively. Each line or circle represents a distinct mouse (*n* = 4–10 mice per OSN subtype and condition). Related to Fig. 3.

bioRxiv preprint doi: https://doi.org/10.1101/2024.02.10.579748; this version posted February 12, 2024. The copyright holder for this preprint (which was not certified by peer review) is the author/funder, who has granted bioRxiv a license to display the preprint in perpetuity. It is made available under aCC-BY 4.0 International license.

Supplementary Figure 4

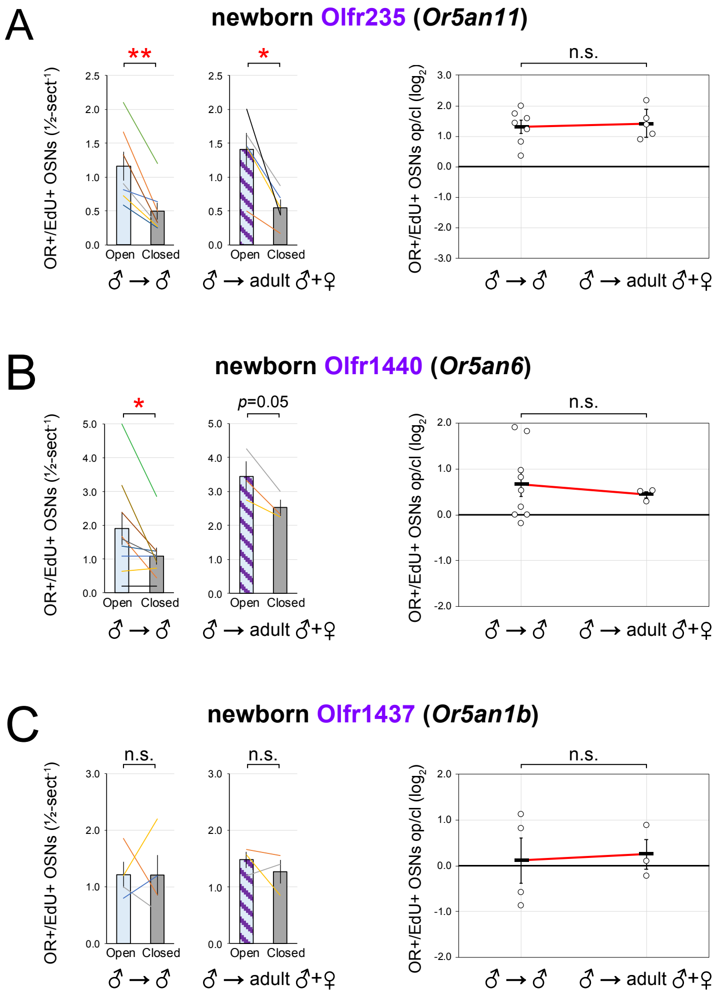

Supplementary Figure 4. UNO-induced changes in quantities of newborn OSNs of specific subtypes as a function of the ages of mice within the odor environment. A–C. Quantification (*left*) and UNO effect sizes (*right*) of newborn OSNs (OR+/EdU+) of subtypes Olfr235 (A), Olfr1440 (B) or Olfr1437 (C) within OEs of UNO-treated male mice exposed to themselves (♂ → ♂) or their parents (♂ → adult ♀ + ♂) at the time of EdU labeling. Musk-responsive subtypes are labeled in purple type. Each line or circle represents a distinct mouse (*n* = 3–9 mice per OSN subtype and condition). Related to Fig. 4.

bioRxiv preprint doi: https://doi.org/10.1101/2024.02.10.579748; this version posted February 12, 2024. The copyright holder for this preprint (which was not certified by peer review) is the author/funder, who has granted bioRxiv a license to display the preprint in perpetuity. It is made available under aCC-BY 4.0 International license.

Supplementary Figure 5

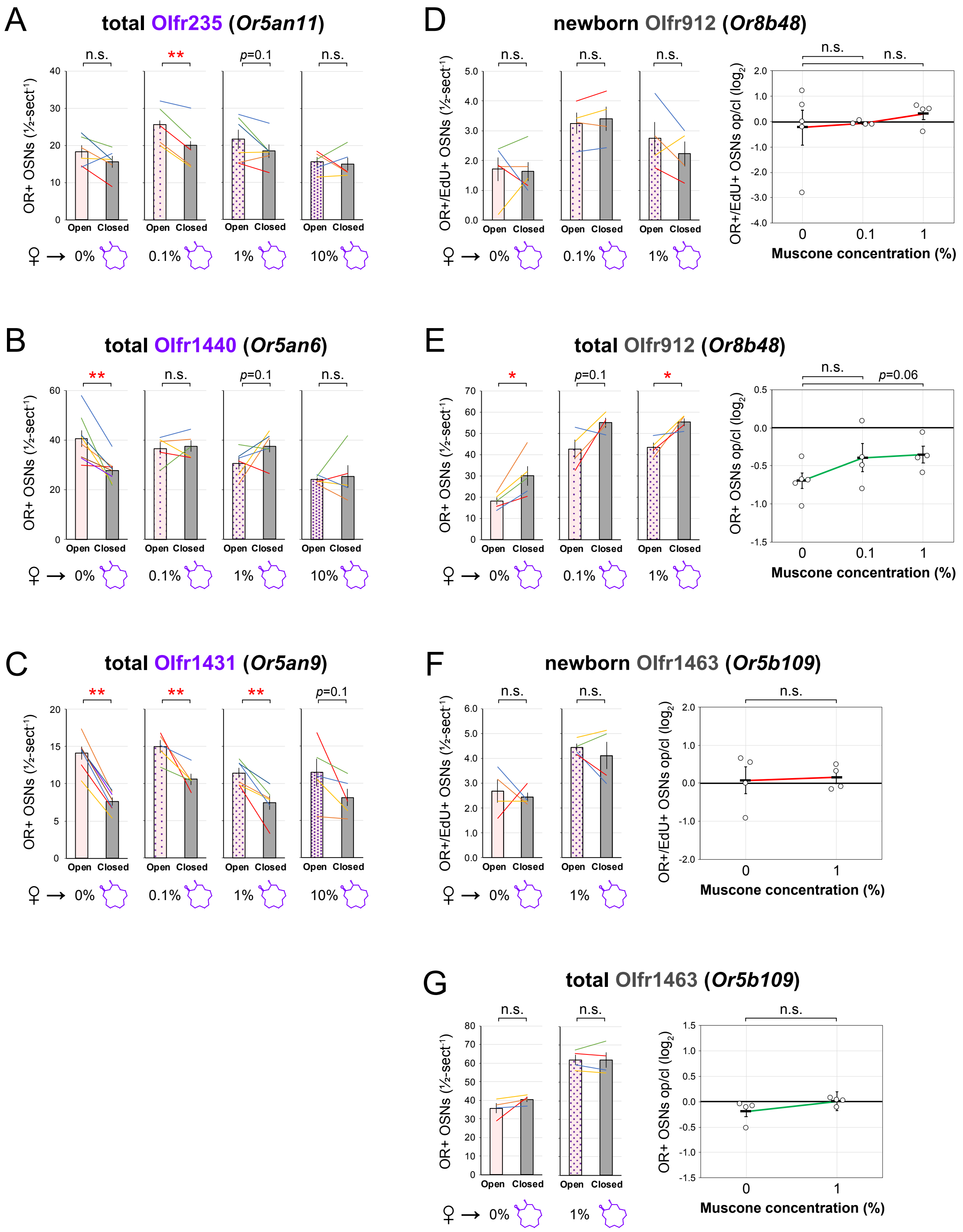

bioRxiv preprint doi: https://doi.org/10.1101/2024.02.10.579748; this version posted February 12, 2024. The copyright holder for this preprint (which was not certified by peer review) is the author/funder, who has granted bioRxiv a license to display the preprint in perpetuity. It is made available under aCC-BY 4.0 International license.

Supplementary Figure 5. Effects of exposure to muscone on UNO-induced changes in the quantities of total and newborn OSNs of specific subtypes. A–C. Quantification of total OSNs (OR+) of subtypes Olfr235 (A), Olfr1440 (B), or Olfr1431 (C) within OEs of UNO-treated female mice exposed to 0, 0.1, 1, or 10% muscone at the time of EdU labeling. D–G. Quantification of (*left*) or UNO effect sizes for (*right*) newborn OSNs (OR+/EdU+) (D, F) or total OSNs (OR+) (E, G) of subtypes Olfr912 (D, E) or Olfr1463 (F, G) within OEs of UNO-treated female mice exposed to varying concentrations of muscone at the time of EdU labeling. Musk-responsive and control subtypes are labeled in purple and gray type, respectively. Each line or circle represents a distinct mouse (n = 5–10 mice per OSN subtype and condition). Related to Fig. 5.

bioRxiv preprint doi: https://doi.org/10.1101/2024.02.10.579748; this version posted February 12, 2024. The copyright holder for this preprint (which was not certified by peer review) is the author/funder, who has granted bioRxiv a license to display the preprint in perpetuity. It is made available under aCC-BY 4.0 International license.

Supplementary Figure 6

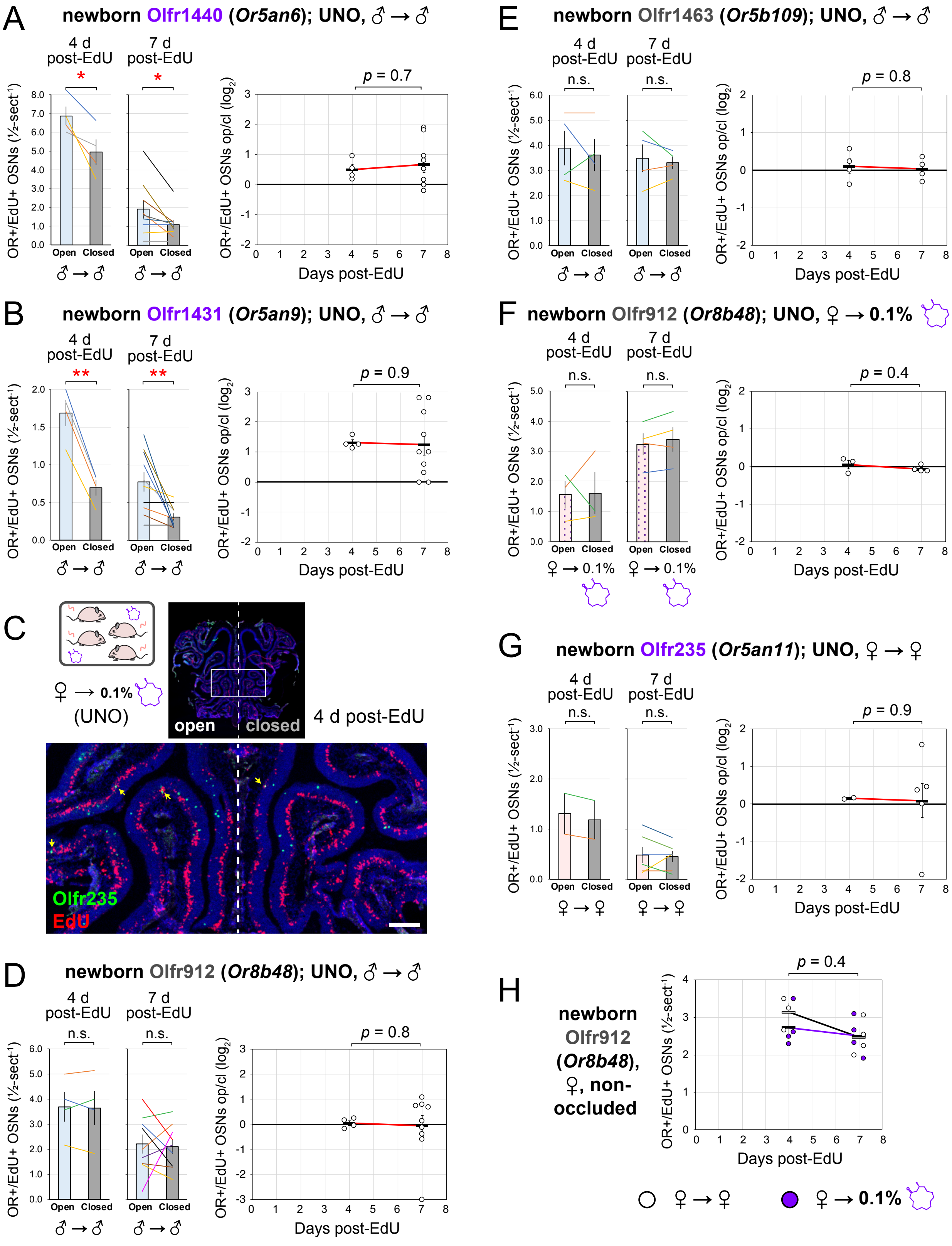

bioRxiv preprint doi: https://doi.org/10.1101/2024.02.10.579748; this version posted February 12, 2024. The copyright holder for this preprint (which was not certified by peer review) is the author/funder, who has granted bioRxiv a license to display the preprint in perpetuity. It is made available under aCC-BY 4.0 International license.

Supplementary Figure 6. Stimulation-dependent changes in newborn OSN quantities are observed immediately after neurogenesis and stable thereafter, consistent with a mechanism involving altered birthrate. A, B, D–G. Quantification of (*left*) and UNO effect sizes for (*right*) newborn Olfr1440 (A), Olfr1431 (B), Olfr912 (D, F), Olfr1463 (E), and Olfr235 (G) OSNs (OR+/EdU+) within OEs of UNO-treated males exposed to themselves (♂ → ♂) (A, B, D, E), females exposed to muscone (♀ → 0.1% muscone) (F), or females exposed to themselves (♀ → ♀) (G) at the time of EdU labeling. C. Representative image of an OE section stained for EdU and Olfr235 mRNA from UNO-treated female mice exposed to muscone (♀ → 0.1% muscone) at the time of EdU-labeling and sacrificed 4 d post-EdU. OR+/EdU+ cells (*yellow arrows*) are newborn Olfr235 OSNs. Scale bar: 150 µm. H. Quantification of newborn (OR+/EdU+) Olfr912 OSNs within OEs of non-occluded females exposed to themselves (♀ → ♀; *black circles*) or to muscone (♀ → 0.1% muscone; *purple circles*) at the time of EdU-labeling. Musk-responsive and control subtypes are labeled in purple and gray type, respectively. Each line or circle represents a distinct mouse (*n* = 2–10 mice per OSN subtype and condition). Related to Fig. 7.

bioRxiv preprint doi: https://doi.org/10.1101/2024.02.10.579748; this version posted February 12, 2024. The copyright holder for this preprint (which was not certified by peer review) is the author/funder, who has granted bioRxiv a license to display the preprint in perpetuity. It is made available under aCC-BY 4.0 International license.

Supplementary Figure 7

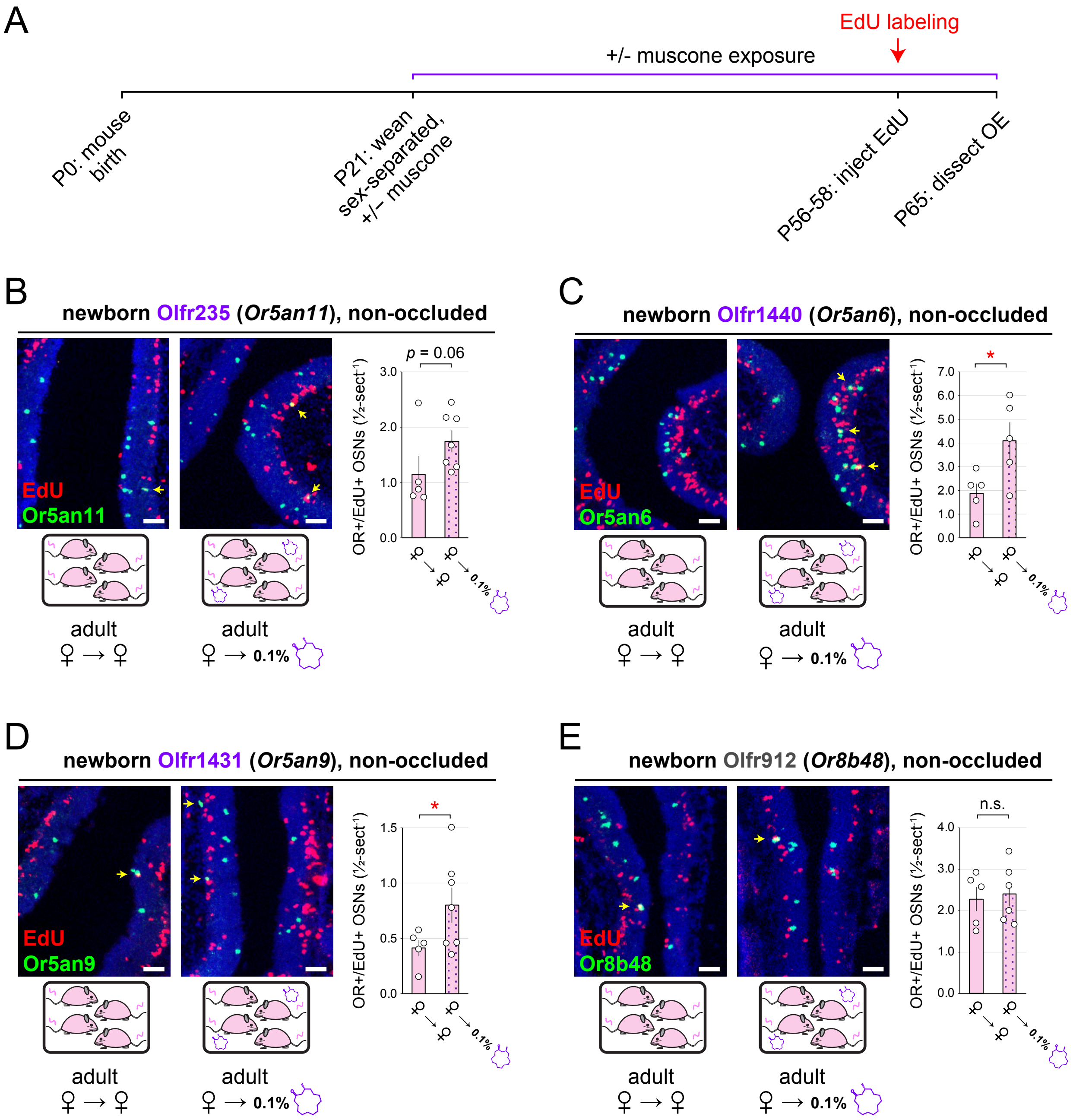

bioRxiv preprint doi: https://doi.org/10.1101/2024.02.10.579748; this version posted February 12, 2024. The copyright holder for this preprint (which was not certified by peer review) is the author/funder, who has granted bioRxiv a license to display the preprint in perpetuity. It is made available under aCC-BY 4.0 International license.

Supplementary Figure 7. Muscone exposure-dependent increases in quantities of newborn OSNs of musk-responsive subtypes persist into adulthood. A. Experimental timeline for the quantification of newborn OSNs of specific subtypes in non-occluded adult mice that were weaned sex-separated with or without muscone exposure starting at P21, EdU-labeled at P56–58, sacrificed at P65, and analyzed *via* OR-specific FISH and EdU staining. B–E. Representative images (*left*) of OE sections stained for EdU and Olfr235 (B), Olfr1440 (C), Olfr1431 (D), or Olfr912 (E) mRNAs, and quantification (*right*) of newborn (OR+/EdU+) Olfr235 (B), Olfr1440 (C), Olfr1431 (D), or Olfr912 (E) OSNs within OEs of non-occluded females exposed to themselves (♀ → ♀) or to muscone (♀ → 0.1% muscone) at the time of EdU-labeling. OR+/EdU+ cells (*yellow arrows*) are newborn Olfr235 OSNs. Scale bars: 150 µm. Musk-responsive and control subtypes are labeled in purple and gray type, respectively. Each circle represents a distinct mouse (*n* = 5–7 mice per OSN subtype and condition).

bioRxiv preprint doi: https://doi.org/10.1101/2024.02.10.579748; this version posted February 12, 2024. The copyright holder for this preprint (which was not certified by peer review) is the author/funder, who has granted bioRxiv a license to display the preprint in perpetuity. It is made available under aCC-BY 4.0 International license.

Supplementary Table 1. Summary of animals used.

Condition	Age of UNO treament	Period of muscone exposure	Age of EdU treament	Age of OE dissection (days post-EdU)	Muscone concentration (%)	Number of mice used						
						Olfr235 (*Or5an11*)	Olfr1440 (*Or5an6*)	Olfr1431 (*Or5an9*)	Olfr1437 (*Or5an1b*)	Olfr1419 (*Or10q3*)	Olfr912 (*Or8b48*)	Olfr1463 (*Or5b109*)
**Sex-separated males**	P14	-	P28	P32 (4 d)	-	4	4	4	-	-	4	4
				P35 (7 d)		7	9	10	4	6	9	4
	-			P35 (7 d)		10	-	-	-	-	4	-
**Sex-separated females**	P14	-	P28	P32 (4 d)	-	2	-	-	-	-	-	-
				P35 (7 d)		6	8	7	-	-	5	4
	-		P28	P35 (7 d)		10	5	9	-	-	4	-
			P28	P32 (4 d)		3	3	3	-	-	3	-
			P56-P58	P65 (7-9 d)		5	5	5	-	-	5	-
**Sex-combined females**	P14	-	P28	P35 (7 d)	-	7	5	5	-	-	4	4
	-					5	-	-	-	-	4	-
**Adult-exposed males**	P14	-	P28	P35 (7 d)	-	5	3	6	3	3	5	-
**Muscone-exposed females**	P14	P21-P35	P28	P32 (4 d)	0.1	5	5	5	-	-	3	-
				P35 (7 d)	0.1	5	5	5	-	-	4	-
				P35 (7 d)	1	6	6	6	-	-	4	4
				P35 (7 d)	10	5	5	5	-	-	-	-
	-	P21-P35	P28	P35 (7 d)	0.1	5	5	5	-	-	4	-
			P28	P32 (4 d)	0.1	4	4	4	-	-	4	-
		P21-P65	P56-P58	P65 (7-9 d)	0.1	7	5	7	-	-	6	-

bioRxiv preprint doi: https://doi.org/10.1101/2024.02.10.579748; this version posted February 12, 2024. The copyright holder for this preprint (which was not certified by peer review) is the author/funder, who has granted bioRxiv a license to display the preprint in perpetuity. It is made available under aCC-BY 4.0 International license.

Supplementary Table 2. Summary of FISH probes used.

Target (gene)	OE expression zone	Purpose	Gene region targeted	Genomic position relative to CDS start	Forward primer	Reverse primer	Probe length
**Olfr235 (*Or5an11*)**	2	OSN Quantification	5'-UTR	-7217	TCAACAGCATGTTCAGGGGA	GCCTTTTTCTCACCTGGGCT	673
**Olfr1440 (*Or5an6*)**	2	OSN Quantification	CDS	363	GACCGGTATGCTGCCATTTGTA	TTGTACCCTTAGTGGTGACAT	494
**Olfr1431 (*Or5an9*)**	2	OSN Quantification	3'-UTR	1540	ACTGGTGAGCAAGTCATGGT	CAGTGGCCATGCTTATCAATGT	547
**Olfr1437 (*Or5an1b*)**	3	OSN Quantification	3'-UTR	1190	TTCAGCCCTTGGTCTTGTCC	CCTGACGGTGAGAAGTGTCC	753
**Olfr1419 (*Or10q3*)**	3	OSN Quantification	CDS	33	TTCCACTTCCGCCCCTTTTC	ACTTCTTCTGCGACATGCCT	753
**Olfr912 (*Or8b48*)**	3	OSN Quantification	CDS/3'-UTR	483	TGCATCCTGAGACTGACTTTCT	GCTGTGTCAAATGCAAGATTTTAAA	500
**Olfr1463 (*Or5b109*)**	2	OSN Quantification	CDS	133	GGGTTGATTCTGCTGATTCTTT	GAGCAAAGAAGAGAGTGAAGCTG	502
S100a5	1-4	UNO efficiency analysis	CDS	1	CTTCTTCCTTGGGTACATATCGTT	TGGAAGGATCCATCTGGGAGA	404
